# GWAS-Identified SNPs and Candidate Genes Influencing Sex in Loach (*Misgurnus anguillicaudatus*)

**DOI:** 10.3390/ani16030524

**Published:** 2026-02-06

**Authors:** Junxiao Su, Han Zheng, Yumei Xiang, Yu Zeng, Can Chen, Xiaoyun Zhou, Kaijian Wei

**Affiliations:** 1 College of Fisheries, Huazhong Agricultural University, Wuhan 430070, China; sujx@webmail.hzau.edu.cn (J.S.); zh19@webmail.hzau.edu.cn (H.Z.); chenc111@webmail.hzau.edu.cn (C.C.); 2 Guangxi Aquaculture Introduction and Breeding Center, Nanning 530001, China; xiangyumeia@163.com; 3 Key Laboratory of Southwest China Wildlife Resources Conservation, China West Normal University, Nanchong 637009, China; zengyu_cwnu@163.com

**Keywords:** *Misgurnus anguillicaudatus*, GWAS, sex, SNP, candidate gene

## Abstract

Loach (*Misgurnus anguillicaudatus*) is a small benthic fish favored by consumers in East Asia. Female loaches exhibit greater production value due to their larger size and higher meat yield. Therefore, controlling the sex of loach should be studied to implement an all-female breeding strategy. This study identified sex-related SNP markers and candidate genes in loach through GWAS. Genotyping male and female populations with WGRS revealed 84 SNPs labeled as associated with sex. Distinct SNP peaks appeared on Chr6 and Chr3, with over half of the sex-associated SNPs located on Chr6. Within these sex-associated regions, 15 key candidate genes related to sex and reproduction were identified, including *hemgn* and *foxl2a*. Among these genes, *pik3cb* on Chr3 and *hhip*, *cntln*, and *pard3ba* on Chr6 have multiple sex-associated haplotypes that can be utilized for subsequent monosex breeding. Notably, a highly male-linked SNP marker was identified on *pard3ba*, whose genotype is consistent with the male heterogamete (XX/XY). Through gene enrichment analysis and expression validation, signaling pathways such as TGF-β, FoxO, and mTOR were identified as being involved in sex regulation. This study provides molecular markers for sex-controlled breeding in loach, facilitating in-depth research into the mechanisms of sex regulation in loach.

## Simple Summary

Loach (*Misgurnus anguillicaudatus*) has high nutritional value and is one of the important edible freshwater fish in East Asia. In particular, female loach is popular with consumers because of its large size. Thus, genetic improvements in all-female breeding of loaches better aligns with market demands. Elucidating the mechanism of sex regulation is critical to achieving monosex breeding in aquatic animals. However, the genetic basis for controlling the sex of loaches remains unclear at present. This study performed a genome-wide association study on male and female loaches, identifying sex-associated SNPs and important candidate genes. Notably, a sex-specific SNP marker that effectively identifies the genetic sex of loaches was developed. These findings will advance all-female loach breeding to enhance cultivation efficiency and economic value.

## 1. Introduction

Aquaculture has become a significant source of high-quality animal protein and is crucial for ensuring global nutritional security [1]. Among the many factors that influence aquaculture productivity, sex is particularly important for genetic improvement and production efficiency in aquatic animals. Certain main cultured aquatic animals exhibit clear sexual dimorphism in terms of their economic traits [2,3,4,5]. Given the production advantages of female or male aquatic animals, precise control of sex and breeding of monosex populations has become an essential strategy for improving aquaculture efficiency.

Among anamniotes, including bony or cartilaginous fish, sex has traditionally been thought to depend on a small number of “dominant sex-determining genes”. Notable examples include the sex chromosome-linked duplications genes *dmy*, *amhy*, and *sox3y* [6,7,8,9]. However, with advances in precise genome sequencing and massive population research, sex determination for some animals may result from the combined effects of genetic variants across the genome [10,11,12,13]. In particular, single-nucleotide polymorphisms (SNPs) and insertion–deletion markers (Indels) may regulate the spatiotemporal expression of genes implicated in male and female differentiation [14,15,16,17]. Therefore, identifying and thoroughly investigating sex-associated genetic variations across the entire genome is crucial.

Genome-wide association studies (GWASs) are among the most powerful tools for investigating the mechanisms underlying the development of productive traits within aquaculture animals. Their strength lies in the capacity to comprehensively screen genetic variants and efficiently identify genes strongly associated with target traits [18,19]. Such traits include growth [20,21], body coloration [22], disease immunity [23], feed conversion efficiency [5], swimming ability [24], and environmental tolerance [25,26,27]. Particularly in sex-related studies, GWAS can also identify sex-specific molecular markers for screening pseudo-male fish or supermale fish to assist in producing monosex populations [28,29].

Asia is the most rapidly expanding region in the world when it comes to aquaculture [30]. The loach (*Misgurnus anguillicaudatus*) is a small benthic fish and is an important aquaculture species in East Asia. Female loaches exhibit greater production value due to their significant sexual dimorphism in growth [31,32,33]. Therefore, the focus has increasingly been on understanding the mechanisms that control sex in loaches to support the breeding of all-female populations. In this work, GWAS was employed to systematically detect sex-related genetic variants in loach and discover relevant molecular markers and candidate genes. This research provides foundational data for elucidating sex regulation in loach and contributes to achieving all-female breeding.

## 2. Materials and Methods

### 2.1. Ethical Statement

This work followed the “Guidelines for the Conservation and Use of Laboratory Animals of the Hubei Laboratory Animal Care Committee of China”. All experimental operations involving fish were permitted by the Laboratory Animal Ethics Committee of Huazhong Agricultural University (Permit Number: HZAUFI-2018-026; 27 March 2018). All fish were anesthetized with MS-222 (100 mg/L) before the experiment.

### 2.2. Sample Collection and Processing

Sixty sexually mature wild loaches (thirty of each sex) were obtained from three locations in the Yangtze River Basin, Huanggang (HG), Wuhan (WH), and Suizhou (SZ), for whole-genome resequencing (WGRS) and GWAS. Specifically, the HG population comprised 13 males and 13 females, the WH population had 5 males and 5 females, and the SZ population included 12 males and 12 females. All fish were confirmed as diploid using flow cytometry (Becton Dickinson Biological Sciences, Franklin Lakes, NJ, USA). Before tissue collection, fish were fasted for 48 h in still indoor water. Muscle and partial gonad tissues were cryopreserved in −80 °C for DNA and RNA isolation, respectively. The remaining gonads were fixed in Bouin’s solution for 24 h, dehydrated with a gradient of ethanol solutions, and then embedded in paraffin. The gonads were subsequently sectioned into 5 μm sections, stained with hematoxylin and eosin, and mounted for histological observation. The physiological sex of all individuals was confirmed histologically before sequencing and analysis.

To observe gonadal development, diploid loaches were artificially bred in our laboratory. Hatching of fertilized eggs and rearing of larvae were conducted in aerated water at 25 ± 0.5 °C under 100 lx illumination (12 h light/12 h dark). The larvae were fed *Artemia* twice a day (9:00 and 18:00). When larval loaches grew to juveniles (30 ± 2 mm), they were transferred to outdoor ponds and fed commercial loach feed (crude protein content 42%) twice daily (9:00 and 18:00). After completion of the primary gonadal differentiation stage, six males and six females were randomly selected each month for histological observation to determine the gonadal development process.

### 2.3. Genomic DNA Extraction, Genotyping, and SNP Annotation

Using a commercial kit (Cret-Biotech, Suzhou, China), the muscle tissue of the loaches was fully ground and lysed with protease K, and the genomic DNA was isolated and purified using magnetic beads. Genomic DNA from 60 loaches was used for library construction and subsequently sequenced on the Illumina HiSeq 2500 platform. The original sequencing underwent stringent quality control, including removing adapter sequences, trimming bases (mean phred score < 20), and excluding undetected bases (N bases > 10). Filtered reads were mapped to the diploid loach reference genome (NCBI registration: GCF_027580225.1) by BWA-MEM v1.0.6, and SNP calling was performed after SAMTOOLS 1.15.1 removal of duplication [34,35]. GATK v4.3.0.0 retained high-confidence SNPs with minor allele frequency (MAF) > 0.05, missing data rate < 30%, and an average site depth > 4×. SNPs were functionally annotated by SNPEff 5.1d software in combination with gene prediction information from the reference genome [36].

### 2.4. Population Structure and Linkage Disequilibrium (LD) Analysis

Principal component analysis (PCA) of pruned genotypes with PLINK v1.90 b6.20, as well as visualized genetic relationships of loaches based on the first three principal components (3PC) [37]. A phylogenetic tree was gained using the neighbor-joining (NJ) method with FastTree 2.1.11, and simulations were run 1000 times [38]. Population structure was deduced with Admixture v1.3.0, and the most suitable number of ancestral populations (K) was identified according to the minimum cross-validation (CV) error [39].

The squared correlation coefficient (r^2^) was computed among any two SNPs within 500 kb by PopLDdecay v3.41 [40]. The physical distance for r^2^ decayed halfway to its maximum size was the LD half-decay distance.

### 2.5. Genome-Wide Association Study

Sex was encoded as a binary variable, with phenotypic females and males assigned as 1 and 2, respectively. GWAS was implemented by the ‘rMVP’ package with both the Fixed and Random Model Circulating Probability Unification (FarmCPU) approaches and General Linear Model (GLM) (Supplementary Table S1) [41]. As covariates in the models, we utilized 3PC and the kinship matrices. The GLM equation is expressed as follows:

*y* = *Xα* + *Zβ* + *e*  (1)

where *y* is the phenotype, and *X* represents the indicator matrix of the fixed effect with parameters *α*; *Z* is the indicator matrix of the SNP, with effect *β*; *e* is random error, which obeys *e* ∼ (0, *σ*_*e*_^2^). Associations between SNPs and sex were calculated using the ‘rMVP’ package. The significance threshold according to the Bonferroni correction method was *p*-value < 3.22 × 10^−9^ (0.05/number of SNPs). However, SNPs with production value are difficult to obtain. To investigate more SNPs associated with sex, the genome-wide threshold was set at *p*-value < 1 × 10^−6^ according to Wu’s method for identifying sex-associated SNPs (Supplementary Table S2) [42]. The final sex-specific SNP marker was validated. SNP density plots and Manhattan plots were drawn using the CMplot package in RStudio v4.2.2, while quantile–quantile (QQ) plots were generated with the ‘qqman’ package.

### 2.6. LD Haplotype Association Analysis

LD heatmaps for significant SNP-enriched regions were drawn with LDBlockShow v.1.40. LD blocks were identified based on an R^2^ threshold of 0.8. Haplotypes comprising significantly associated SNPs and nearby SNPs were visualized by Haploview v.4.2. Genotype proportions were calculated for each haplotype by physiological sex in the sequenced population. Fisher’s exact test was applied to analyze significant differences between males and females (*p* < 0.05).

### 2.7. Candidate Gene Annotation and Functional Enrichment

For each significant sex-associated SNP in the loach reference genome, 50 kb flanking regions were extracted as sex-associated candidate intervals [43]. Protein-coding genes within these intervals were annotated through UniProtKB, Swiss-Prot, and NCBI databases (Supplementary Table S3). Gene Ontology (GO) and Kyoto Encyclopedia of Genes and Genomes (KEGG) enrichment analyses were conducted with the R function ‘ClusterProfiler’. Terms with *p*-values < 0.05 were regarded as enriched. The results of enrichment analyses were visualized using an online platform [44]. Protein–protein interaction (PPI) predictions for sex-biased expression candidate genes were constructed by Cytoscape v.3.6.1, according to the STRING database (https://string-db.org, accessed on 14 May 2025). PPI networks refer to the results reported in zebrafish.

### 2.8. The mRNA Expression of Important Candidate Genes

Gonadal tissues from three sexually mature female and male loaches were used for total RNA isolation. First-strand cDNA was synthesized by reverse transcription of total RNA after removal of genomic DNA (TaKaRa, Kyoto, Japan). Candidate sex-related genes were chosen according to prior functional studies in vertebrates as well as loach genomic variation data. qRT-PCR was performed on an ABI 7300 Real-Time PCR System (Applied Biosystems, Foster, CA, USA) using the TB Green^®^ Premix Ex Taq™ II kit (TaKaRa, Cat. No. RR820A, Kyoto, Japan). Expression levels were normalized and calculated by the $2^{-\Delta \Delta Ct}$ approach. Significant differences between groups were evaluated with a *t*-test (*p* < 0.05). Gene sequence information and primers are provided in Supplementary Tables S4 and S5.

### 2.9. Development of Sex-Specific SNP Markers

We extracted 500 bp flanking sequences of significant sex-associated SNPs using Samtools. Primers specific to these SNPs were designed using Primer 5.0. Validation was performed in three independent populations distinct from those used in GWAS: a laboratory-bred population (population 1, 117 individuals) and two independent cultured populations (populations 2, 84 individuals; populations 3, 88 individuals). SNP loci were PCR-amplified from the Genomic DNA of all individuals using a premix kit (AGcurate, Code No. AG12207, Changsha, China). The PCR amplification procedure included pre-denaturation at 95 °C for 3 min; 94 °C for 25 s, 52 °C for 25 s, 72 °C for 10 s, 35 cycles; and a final extension at 72 °C for 5 min. PCR products were separated on 1% agarose gels and subsequently subjected to Sanger sequencing. The sequencing results were visualized using SnapGene 4.2.4 to distinguish homozygous and heterozygous SNPs based on peak patterns. Gene structures surrounding the SNP loci were illustrated using the online tool (http://wormweb.org/exonintron, accessed on 8 June 2025). Primers for sex-specific SNP marker are provided in Supplementary Table S5.

## 3. Results

### 3.1. Phenotypic Analysis

**Figure 1:**
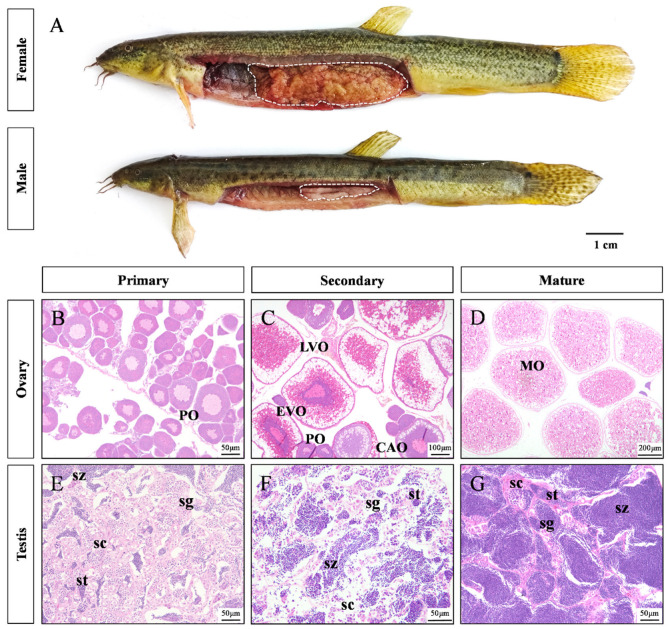
Morphological and gonadal differences between male and female loach. (**A**) Body size and gonadal anatomy of both female and male individuals. Gonadal outlines are indicated by white dashed lines. (**B**–**D**) Ovarian development process. (**E**–**G**) Testis development process. PO: primary oocyte; CAO: cortical–alveolar oocyte; EVO: early vitellogenic oocyte; LVO: late vitellogenic oocyte; MO: mature oocyte; sg: spermatogonia; sc: spermatocytes; st: spermatids; sz: spermatozoa. Scale bars: (**A**), 1 cm; (**B**,**E**–**G**), 50 μm; (**C**), 100 μm; (**D**), 200 μm.

Sexually mature female loaches exhibit significantly larger bodies than males (Figure 1A). To investigate the basis of this sexual dimorphism, gonadal development was continuously observed histologically in loaches. Under the influence of both genetic and environmental factors, primordial germ cells (PGCs) proliferate through successive mitotic and meiotic divisions, differentiating into primary oocytes in females and spermatogonia or primary spermatocytes in males (Figure 1B,E). As development progresses into the secondary gonadal stage, oocytes and spermatocytes continue meiosis (Figure 1C,F). Eventually, the ovaries and testes become filled with mature oocytes and spermatozoa, respectively (Figure 1D,G).

### 3.2. Sequencing and Population Structure Analysis

**Figure 2:**
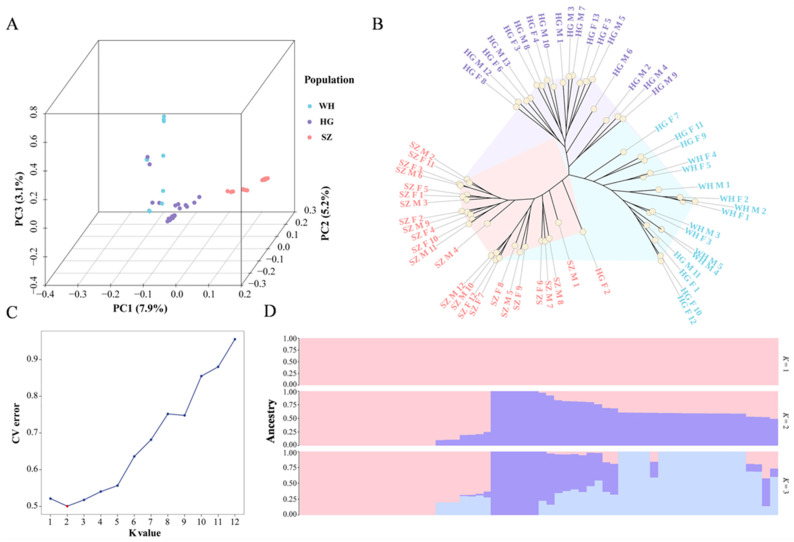
Genetic structure and phylogenetic relationships among the studied population. (**A**) 3D PCA showing the genetic clustering of individuals from different populations. (**B**) NJ phylogenetic tree based on genome-wide SNPs. Samples sharing the same color and region label are clustered within the same branch. (**C**) Cross-validation error rate of K = 1–12. The point with the smallest error is marked in red. (**D**) Population structure plots for K = 1 to 3. Each color indicates a distinct ancestral genetic component.

WGRS was conducted on 60 loach individuals from three different geographic populations, producing high-quality data. The average alignment rate to the reference genome was 97.44%, with a mean sequencing depth of 19.75× and genome coverage of 92.22%. PCA indicated the loaches mainly aggregated into three groups corresponding to their geographic regions, though a few HG individuals grouped with the WH population (Figure 2A). Similarly, the NJ phylogenetic tree indicates that SZ individuals formed an independent branch, except one HG individual, while most HG individuals formed a second branch (Figure 2B). The WH population and a few HG individuals constituted a third branch. Population structure analysis revealed that K = 2 is the optimal number, suggesting that these loach populations likely originated from at least two ancestral lineages (Figure 2C,D). These results indicate that the loach samples selected for sequencing and analysis reduced potential errors arising from population differentiation.

### 3.3. Genetic Background Information

**Figure 3:**
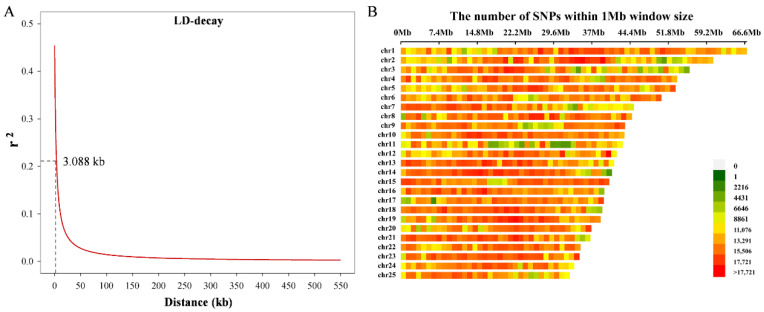
Genetic variation landscape of the studied population. (**A**) LD decay plot. The physical distance between SNPs is shown on the *x*-axis, and *r*^2^ value on the *y*-axis. The dashed line marks the half-decay. (**B**) Distribution and density of high-confidence SNPs among the 25 chromosomes.

After stringent quality control and filtering, 15,517,503 high-quality SNPs were retained for use in GWAS. Linkage disequilibrium showed rapid decay, indicating frequent recombination within the loach populations. The maximum *r*^2^ value was 0.454, and the physical distance at LD half-decay was 3.088 kb (Figure 3A). The average SNP density across the 25 chromosomes (Chr) was approximately one SNP per 79.55 bp, and SNPs were unevenly distributed among chromosomes (Figure 3B). For instance, Chr1 contained the largest number of SNPs (840,972), whereas Chr23 exhibited the highest average SNP density (one SNP per 72.22 bp).

### 3.4. GWAS Analysis

Given the high recombination rate within loach populations, both the FarmCPU and GLMs were used to conduct the GWAS for sex, incorporating the top 3PC as covariates, to minimize false positives and improve detection power. Notably, the significant SNPs as determined by the FarmCPU and GLM methods were identical when under the threshold of *p* < 10^−6^, as detailed in Supplementary Table S1. Subsequent results are presented based on the FarmCPU model.

**Figure 4:**
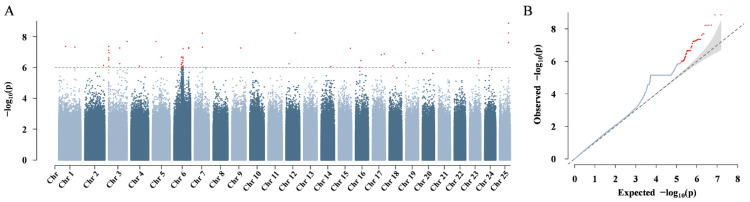
GWAS results associated with sex. (**A**) Manhattan plot generated using the FarmCPU model. The dotted horizontal line denotes the genome-wide cutoff for significance. The red dots represent SNPs significantly associated with sex. The dark/light blue dots represent non-significant SNPs associated with sex. (**B**) Q-Q plot of the FarmCPU analysis. The *x*-axis represents the expected −log_10_(*p*) values under the null hypothesis, while the *y*-axis displays the observed −log_10_(*p*) values for each SNP. The dashed diagonal line (slope = 1) represents the theoretical distribution in the absence of association. The red dots represent SNPs significantly associated with sex. The blue dots represent non-significant SNPs associated with sex. The shaded area represents the 95% confidence interval.

A total of 84 SNPs were labeled as significantly associated with sex, with effect sizes ranging from −0.81 to 0.83, as detailed in Supplementary Table S2. Nine of these SNPs mapped to the coding regions of genes such as *klf5a*, *znf271*, *dnajc16*, and several uncharacterized genes. A further 52 SNPs were mapped to the introns of genes, including *pik3cb*, *atm*, *hhip*, *dis3*, *bnc2*, *cntln*, *esco1*, and *pard3ba*, while two SNPs were detected in the 3′ UTRs of *hhip* and *klf5a*. The remaining 21 SNPs were intergenic. Notably, a strong association SNP peak was observed on Chr6, accounting for over half of all significant SNPs (43 in total) (Figure 4A). Furthermore, 13 SNPs were identified on Chr3. The quantile–quantile (Q-Q) plot verified the reliability of the associated SNPs recognized from FarmCPU (Figure 4B).

### 3.5. Haplotype Analysis of Genes Enriched in Significantly Associated SNPs

**Figure 5:**
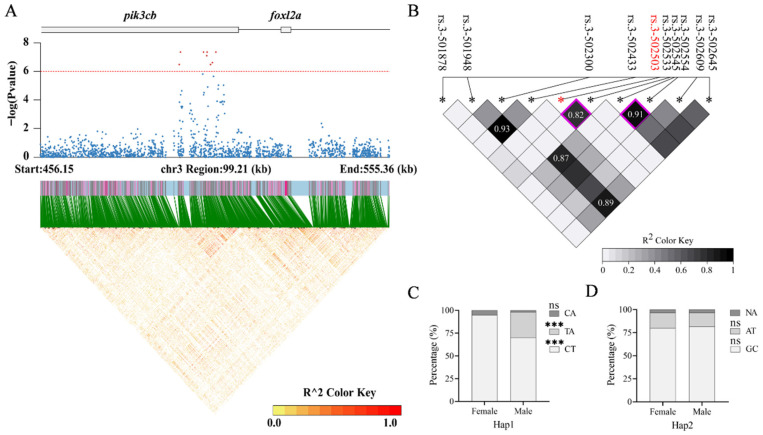
Haplotype analysis of *pik3cb* on Chr3. (**A**) Regional association plot with LD heatmap on Chr3. The gene arrangement based on genome annotation is displayed at the top of the figure, with the gene and intergenic regions represented by boxes and lines, respectively. Red dots represent SNPs significantly associated with sex. Blue dots represent non-significant SNPs associated with sex. The red dotted line represents the significance threshold. (**B**) LD heatmap for a significantly associated SNP in *pik3cb*. Red font and asterisk marked significantly associated SNP; black fonts and asterisks marked nearby SNPs. When the R^2^ between the two SNPs is ≥0.8, the block shows its specific value. Squares with a purple border represent haplotypes formed by adjacent SNPs. (**C**,**D**) The percentage of genotypes for the two haplotypes in male and female populations. The “***” above the genotype indicates a significant difference in its proportion between males and females (*p* < 0.001). ns: no statistical difference (*p* > 0.05).

**Figure 6:**
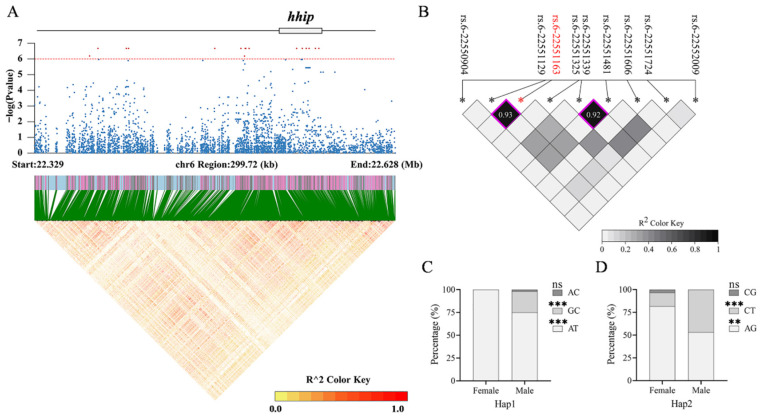
Haplotype analysis of *hhip* on Chr6. (**A**) Regional association plot with LD heatmap on Chr6. Red dots represent SNPs significantly associated with sex. Blue dots represent non-significant SNPs associated with sex. The red dotted line represents the significance threshold. (**B**) LD heatmap of significantly associated SNP in *hhip*. Red font and asterisk marked significantly associated SNP; black fonts and asterisks marked nearby SNPs. Squares with a purple border represent haplotypes formed by adjacent SNPs. (**C**,**D**) The percentage of genotypes for two haplotypes in male and female populations. The “***” above the genotype indicates a significant difference in its proportion between males and females (*p* < 0.001). The “**” indicates *p* < 0.01. ns: no statistical difference (*p* > 0.05).

**Figure 7:**
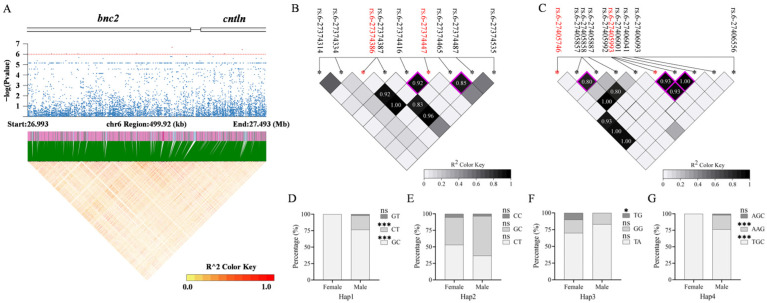
Haplotype analysis of *cntln* on Chr6. (**A**) Regional association plot with LD heatmap on Chr6. Red dots represent SNPs significantly associated with sex. Blue dots represent non-significant SNPs associated with sex. The red dotted line represents the significance threshold. (**B**,**C**) LD heatmaps of significantly associated SNPs in *cntln*. Red fonts and asterisks marked significantly associated SNP; black fonts and asterisks marked nearby SNPs. Squares with a purple border represent haplotypes formed by adjacent SNPs. (**D**–**G**) The percentage of genotypes for four haplotypes in male and female populations. The “***” above the genotype indicates a significant difference in its proportion between males and females (*p* < 0.001). The “*” indicates *p* < 0.05. ns: no statistical difference (*p* > 0.05).

Further analysis revealed that three genomic regions were clustered with significantly sex-associated SNPs. Specifically, the regions were as follows: Chr3: 495588–506044 (10.46 kb); Chr6: 22374410–22564913 (190.50 kb); Chr6: 27043452–27443291 (399.83 kb). They contained eight, sixteen, and eleven SNPs, respectively. Genome annotation indicated that all eight SNPs on Chr3 were located within introns of *pik3cb* (Figure 5A). Notably, *foxl2a*, a key regulator of ovarian development, is positioned 13.06 kb upstream of *pik3cb*, suggesting potential regulatory interaction. On Chr6, the first associated region contained only *hhip*, with six SNPs located within its non-coding region (Figure 6A). In the second region on Chr6, six and five SNPs were found within introns of *bnc2* and *cntln*, respectively (Figure 7A). LD heatmaps were created for all genetic markers within these three areas and their flanking 50 ± 5 kb regions. No large-scale haplotype blocks (R^2^ > 0.8) were identified in these regions, indicating that recombination events occur frequently within them (Figure 5A, Figure 6A and Figure 7A).

Subsequent analyses examined the linkage between the significant SNP loci and their surrounding genetic variations in *pik3cb*, *hhip*, *bnc2*, and *cntln*. No strong LD (R^2^ > 0.8) or haplotype was observed for SNPs in *bnc2* alone. Two haplotypes were identified in *pik3cb,* including *pik3cb*-Hap1 (rs.3-502503, rs.3-502533) and *pik3cb*-Hap2 (rs.3-502545, rs.3-502554) (Figure 5B). Similarly, two haplotypes were found in *hhip*, including *hhip*-Hap1 (rs.6-22551129, rs.6-22551163) and *hhip*-Hap2 (rs.6-22551339, rs.6-22551481) (Figure 6B). Four haplotypes were identified in *cntln*, including *cntln*-Hap1 (rs.6-27374416, rs.6-27374447), *cntln*-Hap2 (rs.6-27374465, rs.6-27374487), *cntln*-Hap3 (rs.6-27405857, rs.6-27405858), and *cntln*-Hap4 (rs.6-27405993, rs.6-27406001, rs.6-27406041) (Figure 7B,C). The genotype proportion for each haplotype was calculated for both the studied male and female populations (Supplementary Table S6). Except for *pik3cb*-Hap2 and *cntln*-Hap2, the genotype proportion of the remaining six haplotypes showed a significant sex bias distribution within the studied population (Figure 5C,D, Figure 6C,D and Figure 7D–G). These six haplotypes can serve as candidate molecular markers to assist the monosex breeding of loach in the future.

### 3.6. Identification and Functional Enrichment of Candidate Genes

As detailed in Supplementary Table S3, 103 known genes and 30 uncharacterized genes were annotated in the regions approximately 50 kb flanking each significantly associated SNP. Several of these genes are involved in sex regulation and reproduction in vertebrates. These include genes affecting spermatogenesis and male reproductive function, such as *spag5*, *hemgn*, *dis3*, *bnc2,* and others, as well as genes implicated in ovarian development, like *foxl2a*, *pspc1*, and *esco1*. Several regulators of signaling pathways involved in gametogenesis were also identified, particularly *pik3cb*, *hhip*, *tgfbr1a*, and *smad1*. Additionally, genes involved in immune responses (*nlrp3*, *aif1l*, *lyve1*) and energy metabolism (*chst10*, *mzt1*, *slc5a7a*) were also represented.

**Figure 8:**
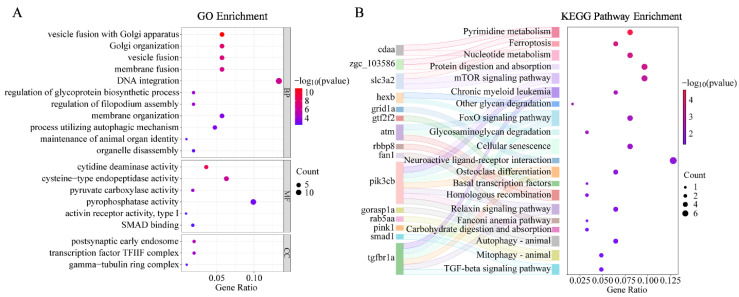
Functional enrichment of candidate genes. (**A**) Top 20 significantly enriched GO terms. (**B**) Sanger plot of the top 20 significantly enriched KEGG pathways. The main genes with significant enrichment are listed on the left, with their associated KEGG pathways on the right. Linkage bands indicate the enrichment relationship between genes and signaling pathways.

GO enrichment analyses were performed on the annotated candidate genes. Enriched biological processes included organelle organization, membrane remodeling, and cytoskeletal regulation (e.g., Golgi organization, vesicle fusion, and filopodium assembly) (Figure 8A). In terms of molecular function, the enriched categories were primarily associated with signal transduction and transcriptional regulation, particularly those involved in carbohydrate and lipid metabolism (e.g., pyruvate carboxylase activity and pyrophosphatase activity), as well as sex-related regulatory functions such as SMAD binding and type I activin receptor activity.

KEGG pathway analysis further revealed that these candidate genes are enriched in pathways involved in basal metabolism, energy homeostasis, cell fate and process, and gonadal development (Figure 8B). Notably, the TGF-β, FoxO, and mTOR signaling pathways are involved in regulating the proliferation, differentiation, and apoptosis of germ cells in vertebrates. Moreover, *pik3cb* and *tgfbr1a* were recurrently enriched across multiple sex-related pathways, suggesting their potential role as regulatory nodes in gonadal differentiation.

### 3.7. Expression and Interaction of Important Candidate Genes

**Figure 9:**
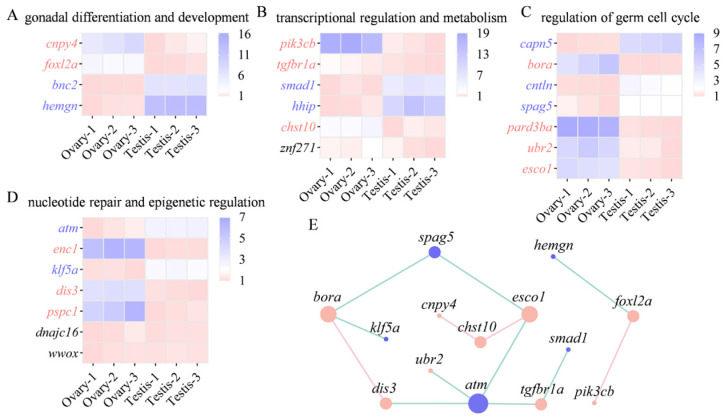
Expression profiles and interaction network of key candidate genes. (**A**) The expressions of genes related to sex differentiation and gonadal development. (**B**) The expressions of genes related to transcriptional regulation and metabolism. (**C**) The expressions of genes related to the regulation of the germ cell cycle. (**D**) The expressions of genes related to nucleotide repair and epigenetic regulation. Each row represents the relative expression level of a single gene between testes and ovaries. Genes that are significantly highly expressed in the ovaries or testes are highlighted in light red or blue, respectively (*p* < 0.05). (**E**) PPI network of genes significantly differentially expressed in testes and ovaries. Node sizes reflect the number of interacting partners. The blue and light red nodes represent the genes with significantly higher expression in the testis and ovary, respectively.

Based on the functional enrichment and genomic variation, 24 candidate genes were prioritized for further investigation. These genes were classified according to their potential functions to assess sex-biased expression in loach gonads. Those involved in sex differentiation and gonadal development showed sex-specific expression profiles: *hemgn* and *bnc2* were expressed at higher levels in testes, while *foxl2a* and *cnpy4* exhibited higher expression in ovaries (*p* < 0.05) (Figure 9A). Within the group associated with transcriptional regulation and metabolism, *pik3cb*, *chst10* and *tgfbr1a* showed higher expression levels in ovaries, whereas *hhip* and *smad1* were significantly higher in testes (*p* < 0.05) (Figure 9B). Most genes associated with the regulation of germ cell cycle, except *capn5*, *cntln* and *spag5*, were highly expressed in ovaries (*p* < 0.05) (Figure 9C). Among the genes associated with nucleotide repair and epigenetic regulation, *atm* and *klf5a* showed higher expression in testes, while *enc1*, *dis3,* and *pspc1* were highly expressed in ovaries (*p* < 0.05) (Figure 9D).

Among the candidate genes differentially expressed between testes and ovaries, *hemgn* and *foxl2a* may interact functionally (Figure 9E). They may operate independently of the regulatory network that maintains germ cell division (*spag5*, *bora*, *esco1*). Together with previous studies in vertebrates, 15 important candidate genes closely related to sex and reproduction were eventually identified (Table 1).

### 3.8. Development of Sex-Specific Molecular Marker

**Figure 10:**
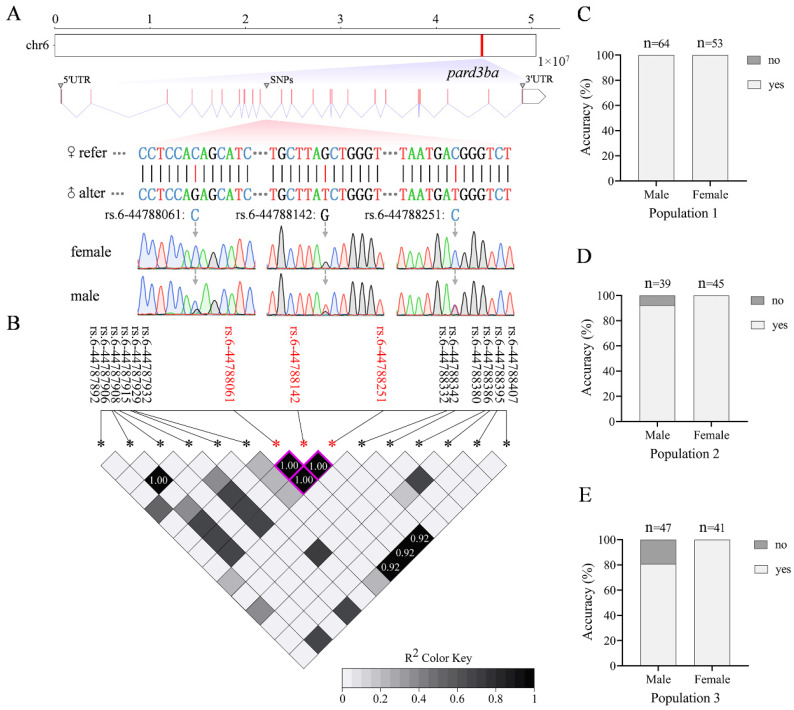
Sex-specific SNP marker. (**A**) The location, sequence, and Sanger sequencing peak map of the sex-specific SNP marker. Red marks the position of *pard3ba* on Chr6. 1 × 10^7^ stands for 10 million bp. Red bars represent exons, and purple folded lines represent introns in the *pard3ba* gene structure. The arrow points to the sequencing peak map corresponding to the mutation site. (**B**) LD heatmap of sex-specific SNP marker. Red fonts and asterisks marked significantly associated SNP; black fonts and asterisks marked nearby SNPs. Squares with a purple border represent haplotypes formed by adjacent SNPs. (**C**–**E**) Identification accuracy of the SNP marker in three loach populations. Male, physiological male; female, physiological female. Markers on the bar graph represent the number of individuals participating in the count. Yes or no represents whether the genetic sex identified by the sex marker corresponds to the physiological sex.

To develop a sex-specific molecular marker to assist with sex-controlled breeding in loach, SNPs significantly associated with sex were screened and verified in males and females through Sanger sequencing (Supplemental Figures S1–S6). The majority of these significant SNPs lie within complex sequence structures, making it difficult to obtain their specific amplification products and stable sequencing peak maps. Nevertheless, a marker combination consisting of three sex-associated SNPs was successfully identified within the intron region of *pard3ba* on chr6: rs.6-44788061, rs.6-44788142, and rs.6-44788251 (Figure 10A). Genotyping revealed that females were homozygous, whereas males were heterozygous at these loci. Notably, the three adjacent SNPs exhibited complete linkage (R^2^ = 1), forming a stable haplotype block that enables reliable identification of the genetic sex (Figure 10B). When tested across three different loach populations, the identification accuracy of this sex-specific SNP marker was 100% (population 1), 92.31% (population 2) and 80.85% (population 3), respectively (Figure 10C–E). These findings demonstrate that this SNP marker can effectively identify the genetic sex in loach.

## 4. Discussion

A thorough understanding of the genetic mechanisms underlying sex determination is essential for accurately implementing sex-controlled breeding [1]. Following advancements in precision sequencing and cellular biotechnology in aquatic animal genetics research, the genetic basis of sex, including the sex chromosome system, main effect genes, and genetic variations, has been progressively studied [45]. The present study identified sex-associated SNP markers and candidate genes via GWAS, which will facilitate deeper perspectives on the molecular basis of sex regulation in loach.

In higher vertebrates, autosomes undergo structural rearrangements to accumulate recombination suppression, eventually evolving into heterogeneous sex chromosomes [46]. By contrast, many lower vertebrates, particularly teleosts, retain sex chromosomes that are morphologically indistinguishable and remain highly similar at the genome sequence [47,48]. Accordingly, SNPs and InDels can serve as effective markers for locating sex chromosomes and sex-determining regions [13,49]. Although the loach is considered to have an XX/XY system, its sex chromosome has not yet been identified [50]. We found that the genotype of the sex molecular marker on Chr6 aligns with the characteristics of male heterogamy in the XY system [47,51]. Furthermore, these sex-associated SNPs were clustered on Chr6, indicating its important role in the genetic sex determination of loach. Notably, 13 significant SNPs were also identified on Chr3, and these signals might not be caused by translocation events or genome assembly errors [52]. Subsequent studies should emphasize more in-depth genomic and functional validation research on Chr6 and Chr3.

It is noteworthy that the genetic sex identified by the sex-specific SNP marker did not fully correspond to the physiological sex in all validation populations. Such inconsistencies are likely due to two factors. Firstly, the physiological sex of most teleosts exhibits high plasticity, with gonadal differentiation being influenced by environmental and endocrine hormones [53]. For example, loach larvae can transform from genetically female to physiologically male in high-temperature environments [54]. Similar mismatches between physiological sex and genetic sex have also been reported in other fishes [47,55,56]. Secondly, substantial genetic differentiation among different geographic populations of loach may affect the accuracy of the sex-specific marker. The lifestyle of loaches limits population dispersal and exacerbates inbreeding, resulting in decreased gene exchange and increased genetic differentiation among populations [57,58,59]. Similar limitations on the cross-population utility of sex-specific markers were also observed in *Cyprinus carpio*, *Takifugu bimaculatus*, and *Collichthys lucidus* [56,60,61].

Genetic variants can strongly affect genes in sex regulatory networks. Variations within the coding regions of crucial genes, including *amhy*, *amhr2,* and *pfpdz1,* can directly alter their role in sex differentiation [9,62,63]. In loach, a sex-associated SNP in the exon of *klf5a*. The homolog of *klf5a*, *klf5*, is crucial for the formation and development of the mouse prostate [64]. Besides coding changes, SNPs in non-coding regions can also be important by modulating transcriptional regulation [65,66]. We identified eight candidate genes that have significant SNPs in introns and showed different expression levels between testes and ovaries in the loach. These genes, including *pik3cb*, *dis3*, *pard3ba*, *esco1*, *atm*, *hhip*, *bnc2,* and *cntln*, along with their homologs, have previously been linked to sex differentiation or reproduction in vertebrates [67,68,69,70,71,72,73,74,75].

However, the reliability of a single SNP may be compromised by recombination. By contrast, haplotypes formed by closely linked SNP aggregation provide more stable and consistent genetic signals. Haplotypes have been utilized in aquaculture to identify genetic markers and regulatory genes for economic traits such as ovarian development, growth, oxygen tolerance, and sex [20,24,63,76]. This study identified multiple haplotypes with a significant sex bias in genotype proportion that were present in *pik3cb*, *hhip*, *cntln*, and *pard3ba*. Taken together, these four genes should be prioritized in future research on the sex and reproduction of loaches.

In vertebrates, sex-determining genes initiate sex differentiation, while male and female regulatory networks dictate the differentiation direction of undifferentiated gonads [77]. Hemgn is a key transcription factor in male sex determination in chickens and has previously been shown to promote male fate by suppressing the expression of *foxl2* during embryonic development [78]. Conversely, *foxl2* is highly conserved across vertebrates and essential for female differentiation and follicle development [79]. Its homolog, *foxl2a*, mainly maintains the development and function of the ovaries in zebrafish [80]. Beyond these sex-determining and differentiation factors, four genes also located in the flanking regions of sex-associated SNPs were implicated in germ cell proliferation and differentiation. Among them, *spag5* is required for spindle integrity during mitosis, while *ubr2* contributes to spermatogenesis through histone ubiquitination [81,82]. In female reproduction, *bora* supports oocyte mitosis by activating Aurora following phosphorylation by Plk1, and *pspc1* regulates oocyte maturation through Chk1 phosphorylation [83,84]. These genes are differentially expressed in testes and ovaries, indicating potential involvement in reproductive regulation in loach. Nonetheless, their specific functions in sex differentiation remain to be experimentally validated.

Sex differentiation and gonadal development are highly coordinated processes that rely on multiple interacting pathways [53,85,86]. TGF-β signaling pathway, which initiates the proliferation and differentiation of PGCs, is one of the most central regulatory pathways in gonadal development in vertebrates [87]. Several of its downstream effectors, such as *amh*, *amhy*, *gsdfy*, *gdf6,* and *gsdf9*, have been demonstrated to be primary effector genes in sex determination across multiple species [88]. In addition, the FoxO and mTOR signaling pathways are involved in regulating gonadal development, especially spermatogonial proliferation and differentiation, as well as spermatogenesis [89,90]. And both pathways are regulated by the PI3K/Akt signaling pathway. These signaling pathways provide important directions for elucidating the molecular basis of gonadal differentiation in loach.

## 5. Conclusions

In the present study, GWAS was utilized to investigate SNPs and candidate genes associated with sex in loach. Of the 84 significantly sex-associated SNPs, over half were clustered on Chr6. Multiple sex-associated haplotypes exhibit a significant sex-biased distribution proportion. Notably, a highly linked sex-specific SNP marker was identified on Chr6, whose genotype is consistent with the male heterogamete (XX/XY). In SNP-associated regions, 15 critical candidate genes were identified, including *hemgn*, *foxl2a*, *dis3*, *esco1*, *bnc2*, *cntln*, and *spag5*. These genes are probably involved in modulating sex differentiation and gamete maturation through the TGF-β, FoxO, and mTOR signaling pathways. Collectively, these insights enhance the understanding of loach sex regulation and contribute to advancing the precision selection breeding of all-female populations of loach.

## Figures and Tables

**Table 1:** Important candidate genes associated with sex and reproduction in *Misgurnus anguillicaudatus*.

Candidate Gene	Significant Expression	Vertebrate Species	Function Verification
*foxl2a*	ovary	*Danio rerio*	maintain ovarian development and function
*bnc2*	testis	*Mus musculus*	main regulator of male germline stem cells
*hemgn*	testis	*Gallus gallus domesticus*	key transcription factors in the process of male sex differentiation
*pik3cb*	ovary	*Oreochromis niloticus*	PI3K-Akt signaling pathway promotes oocyte maturation and ovulation
*hhip*	testis	*Monopterus albus*	significantly up-regulated in female-reversal males
*bora*	ovary	*Xenopus laevis*	*bora* phosphorylation promotes mitosis of germ cells
*cntln*	testis	*Mus musculus*	inactivation of Centlein protein leads to male sterility
*spag5*	testis	*Rattus norvegicus*	crucial for the normal development of testis
*pard3ba*	ovary	*Mus musculus*	*pard3* affects the polarity of Sertoli cells
*ubr2*	ovary	*Mus musculus*	mediate transcriptional silencing during spermatogenesis
*esco1*	ovary	*Sus scrofa domesticus*	knockdown of *esco1* leads to meiotic arrest of oocytes
*atm*	testis	*Mus musculus*	activation of spermatocyte recombination-dependent arrest
*klf5a*	testis	*Mus musculus*	*klf5* is involved in the formation and development of prostate
*dis3*	ovary	*Mus musculus*	maintaining early male germ cell lineage
*pspc1*	ovary	*Mus musculus*	involved in oocyte maturation

Significant expression: comparison of relative expression of candidate genes in the testis and ovary in loach; vertebrate species: research on candidate genes or their homologous genes in vertebrates; function verification: functions of candidate genes or their homologous genes reported in vertebrates.

## Data Availability

The whole-genome resequencing data generated in this study have been submitted to the National Center for Biotechnology Information and will be made open access on 25 February 2026 (NCBI SRA; https://www.ncbi.nlm.nih.gov/sra; accession number PRJNA1414169).

## Supplementary Materials

The following supporting information can be downloaded at https://www.mdpi.com/article/10.3390/ani16030524/s1, Supplementary Table S1: SNPs identified as significantly associated with sex based on GLM and FarmCPU methods; Supplementary Table S2: Specific information on SNPs significantly associated with sex; Supplementary Table S3: Candidate genes associated with sex in *Misgurnus anguillicaudatus*; Supplementary Table S4: Gene sequence information used for qRT-PCR analysis of *Misgurnus anguillicaudatus*; Supplementary Table S5: Primers used for mRNA expression analysis of candidate genes and amplification of sex-specific SNP marker; Supplementary Table S6: Haplotype analysis of SNPs in *pik3cb*, *hhip*, and *cntln*. Supplementary Figure S1: Sanger sequencing map of PCR-amplified products at the sex-specific SNP loci for ten physiological males in population 1. Supplementary Figure S2: Sanger sequencing map of PCR-amplified products at the sex-specific SNP loci for ten physiological females in population 1. Supplementary Figure S3: Sanger sequencing map of PCR-amplified products at the sex-specific SNP loci for ten physiological males in population 2. Supplementary Figure S4: Sanger sequencing map of PCR-amplified products at the sex-specific SNP loci for ten physiological females in population 2. Supplementary Figure S5: Sanger sequencing map of PCR-amplified products at the sex-specific SNP loci for ten physiological males in population 3. Supplementary Figure S6: Sanger sequencing map of PCR-amplified products at the sex-specific SNP loci for ten physiological females in population 3.
