# Supplementary material for: GWAS-Identified SNPs and Candidate Genes Influencing Sex in Loach (Misgurnus anguillicaudatus)
Source: Animals (Basel). 2026 Feb 6;16(3):524. doi: 10.3390/ani16030524 (PMC12896581; doi:10.3390/ani16030524)
Supplement: Supplementary file 1 [file animals-16-00524-s001.zip › animals-4114662-supplementary.pdf]

Supplementary Materials

Supplementary Table S1. SNPs identified as significantly associated with sex based on GLM and FarmCPU methods.

SNP ID	Methods	*P*-value	threshold
rs.1-20849158	GLM	4.22e-08	1.00e-06
	FarmCPU	4.22e-08	
rs.1-48520965	GLM	4.73e-08	1.00e-06
	FarmCPU	4.73e-08	
rs.2-56459438	GLM	7.43e-07	1.00e-06
	FarmCPU	7.43e-07	
rs.3-495588	GLM	3.18e-07	1.00e-06
	FarmCPU	3.18e-07	
rs.3-495952	GLM	4.24e-08	1.00e-06
	FarmCPU	4.24e-08	
rs.3-502503	GLM	4.24e-08	1.00e-06
	FarmCPU	4.24e-08	
rs.3-503456	GLM	7.41e-08	1.00e-06
	FarmCPU	7.41e-08	
rs.3-503515	GLM	4.24e-08	1.00e-06
	FarmCPU	4.24e-08	
rs.3-504516	GLM	3.18e-07	1.00e-06
	FarmCPU	3.18e-07	
rs.3-505043	GLM	2.38e-07	1.00e-06
	FarmCPU	2.38e-07	
rs.3-506044	GLM	4.24e-08	1.00e-06
	FarmCPU	4.24e-08	
rs.3-1161899	GLM	1.04e-07	1.00e-06
	FarmCPU	1.04e-07	
rs.3-1162173	GLM	3.13e-07	1.00e-06
	FarmCPU	3.13e-07	
rs.3-33338471	GLM	5.68e-07	1.00e-06
	FarmCPU	5.68e-07	
rs.3-33338533	GLM	5.38e-08	1.00e-06
	FarmCPU	5.38e-08	
rs.3-55384577	GLM	2.01e-08	1.00e-06
	FarmCPU	2.01e-08	
rs.4-25804581	GLM	8.46e-07	1.00e-06
	FarmCPU	8.46e-07	

SNP ID	Methods	*P*-value	threshold
rs.5-10756236	GLM<br>FarmCPU	2.03e-08<br>2.03e-08	1.00e-06
rs.5-26654404	GLM<br>FarmCPU	2.08e-07<br>2.08e-07	1.00e-06
rs.6-22374410	GLM<br>FarmCPU	6.21e-07<br>6.21e-07	1.00e-06
rs.6-22381361	GLM<br>FarmCPU	2.08e-07<br>2.08e-07	1.00e-06
rs.6-22404945	GLM<br>FarmCPU	2.08e-07<br>2.08e-07	1.00e-06
rs.6-22406773	GLM<br>FarmCPU	2.08e-07<br>2.08e-07	1.00e-06
rs.6-22478477	GLM<br>FarmCPU	2.08e-07<br>2.08e-07	1.00e-06
rs.6-22500163	GLM<br>FarmCPU	2.08e-07<br>2.08e-07	1.00e-06
rs.6-22503147	GLM<br>FarmCPU	6.40e-07<br>6.40e-07	1.00e-06
rs.6-22503742	GLM<br>FarmCPU	2.08e-07<br>2.08e-07	1.00e-06
rs.6-22504186	GLM<br>FarmCPU	2.08e-07<br>2.08e-07	1.00e-06
rs.6-22507005	GLM<br>FarmCPU	2.08e-07<br>2.08e-07	1.00e-06
rs.6-22546477	GLM<br>FarmCPU	2.08e-07<br>2.08e-07	1.00e-06
rs.6-22551163	GLM<br>FarmCPU	2.08e-07<br>2.08e-07	1.00e-06
rs.6-22554439	GLM<br>FarmCPU	2.08e-07<br>2.08e-07	1.00e-06
rs.6-22556800	GLM<br>FarmCPU	2.08e-07<br>2.08e-07	1.00e-06
rs.6-22562044	GLM<br>FarmCPU	2.08e-07<br>2.08e-07	1.00e-06
rs.6-22564913	GLM<br>FarmCPU	2.08e-07<br>2.08e-07	1.00e-06
rs.6-23291805	GLM<br>FarmCPU	9.39e-07<br>9.39e-07	1.00e-06

SNP ID	Methods	*P*-value	threshold
rs.6-23349604	GLM<br>FarmCPU	9.39e-07<br>9.39e-07	1.00e-06
rs.6-23353637	GLM<br>FarmCPU	9.39e-07<br>9.39e-07	1.00e-06
rs.6-24122919	GLM<br>FarmCPU	9.98e-07<br>9.98e-07	1.00e-06
rs.6-24125946	GLM<br>FarmCPU	5.06e-07<br>5.06e-07	1.00e-06
rs.6-25458495	GLM<br>FarmCPU	9.39e-07<br>9.39e-07	1.00e-06
rs.6-26185207	GLM<br>FarmCPU	9.39e-07<br>9.39e-07	1.00e-06
rs.6-26299737	GLM<br>FarmCPU	9.39e-07<br>9.39e-07	1.00e-06
rs.6-26318387	GLM<br>FarmCPU	4.74e-07<br>4.74e-07	1.00e-06
rs.6-27043452	GLM<br>FarmCPU	9.39e-07<br>9.39e-07	1.00e-06
rs.6-27074077	GLM<br>FarmCPU	9.39e-07<br>9.39e-07	1.00e-06
rs.6-27074103	GLM<br>FarmCPU	9.39e-07<br>9.39e-07	1.00e-06
rs.6-27098677	GLM<br>FarmCPU	9.39e-07<br>9.39e-07	1.00e-06
rs.6-27221880	GLM<br>FarmCPU	9.39e-07<br>9.39e-07	1.00e-06
rs.6-27296128	GLM<br>FarmCPU	2.08e-07<br>2.08e-07	1.00e-06
rs.6-27374386	GLM<br>FarmCPU	9.39e-07<br>9.39e-07	1.00e-06
rs.6-27374447	GLM<br>FarmCPU	9.39e-07<br>9.39e-07	1.00e-06
rs.6-27405746	GLM<br>FarmCPU	9.39e-07<br>9.39e-07	1.00e-06
rs.6-27405993	GLM<br>FarmCPU	9.39e-07<br>9.39e-07	1.00e-06
rs.6-27443291	GLM<br>FarmCPU	3.53e-07<br>3.53e-07	1.00e-06

SNP ID	Methods	*P*-value	threshold
rs.6-27672625	GLM<br>FarmCPU	2.43e-07<br>2.43e-07	1.00e-06
rs.6-27771987	GLM<br>FarmCPU	9.03e-07<br>9.03e-07	1.00e-06
rs.6-27782483	GLM<br>FarmCPU	6.26e-08<br>6.26e-08	1.00e-06
rs.6-44745870	GLM<br>FarmCPU	5.15e-08<br>5.15e-08	1.00e-06
rs.6-44788061	GLM<br>FarmCPU	5.58e-08<br>5.58e-08	1.00e-06
rs.6-44788142	GLM<br>FarmCPU	5.58e-08<br>5.58e-08	1.00e-06
rs.6-44788251	GLM<br>FarmCPU	5.58e-08<br>5.58e-08	1.00e-06
rs.7-23831209	GLM<br>FarmCPU	5.92e-09<br>5.92e-09	1.00e-06
rs.7-23831219	GLM<br>FarmCPU	6.01e-09<br>6.01e-09	1.00e-06
rs.7-23831258	GLM<br>FarmCPU	4.87e-08<br>4.87e-08	1.00e-06
rs.9-27437449	GLM<br>FarmCPU	5.39e-08<br>5.39e-08	1.00e-06
rs.12-9314173	GLM<br>FarmCPU	5.80e-07<br>5.80e-07	1.00e-06
rs.12-27641629	GLM<br>FarmCPU	5.78e-09<br>5.78e-09	1.00e-06
rs.14-29442705	GLM<br>FarmCPU	9.09e-07<br>9.09e-07	1.00e-06
rs.15-36268252	GLM<br>FarmCPU	5.59e-08<br>5.59e-08	1.00e-06
rs.16-13240372	GLM<br>FarmCPU	9.66e-07<br>9.66e-07	1.00e-06
rs.16-16389188	GLM<br>FarmCPU	3.49e-07<br>3.49e-07	1.00e-06
rs.17-27400821	GLM<br>FarmCPU	1.47e-07<br>1.47e-07	1.00e-06
rs.17-36307674	GLM<br>FarmCPU	1.30e-07<br>1.30e-07	1.00e-06

SNP ID	Methods	*P*-value	threshold
rs.18-12110368	GLM	8.17e-07	1.00e-06
	FarmCPU	8.17e-07	
rs.19-110807	GLM	4.85e-07	1.00e-06
	FarmCPU	4.85e-07	
rs.20-1485091	GLM	1.15e-07	1.00e-06
	FarmCPU	1.15e-07	
rs.20-31965512	GLM	7.75e-08	1.00e-06
	FarmCPU	7.75e-08	
rs.23-28457737	GLM	3.54e-07	1.00e-06
	FarmCPU	3.54e-07	
rs.23-28458362	GLM	6.65e-07	1.00e-06
	FarmCPU	6.65e-07	
rs.25-27346992	GLM	1.30e-09	1.00e-06
	FarmCPU	1.30e-09	
rs.25-27347225	GLM	1.30e-09	1.00e-06
	FarmCPU	1.30e-09	
rs.25-27349254	GLM	2.43e-08	1.00e-06
	FarmCPU	2.43e-08	
rs.25-27349773	GLM	5.78e-09	1.00e-06
	FarmCPU	5.78e-09	

SNP ID, SNPs are denoted by chromosome and physical position; *P*-value, the association between SNPs and sex under two methods, respectively; threshold, genome-wide association threshold.

Supplementary Table S2. Specific information on SNPs significantly associated with sex.

SNP ID	Chr	Position	Allele	Effect	Location
rs.1-20849158	1	20849158	T/G	0.77	intronic (*capn5*)
rs.1-48520965	1	48520965	T/C	0.77	intergenic
rs.2-56459438	2	56459438	G/T	0.37	intronic (*znf845*)
rs.3-495588	3	495588	T/G	0.74	intronic (*pik3cb*)
rs.3-495952	3	495952	A/G	0.78	intronic (*pik3cb*)
rs.3-502503	3	502503	T/C	0.78	intronic (*pik3cb*)
rs.3-503456	3	503456	T/C	0.74	intronic (*pik3cb*)
rs.3-503515	3	503515	A/G	0.78	intronic (*pik3cb*)
rs.3-504516	3	504516	T/C	0.74	intronic (*pik3cb*)
rs.3-505043	3	505043	T/C	0.68	intronic (*pik3cb*)
rs.3-506044	3	506044	C/A	0.78	intronic (*pik3cb*)
rs.3-1161899	3	1161899	A/T	0.74	intronic (*atm*)
rs.3-1162173	3	1162173	C/G	0.74	intronic (*atm*)
rs.3-33338471	3	33338471	C/G	0.77	intergenic
rs.3-33338533	3	33338533	T/C	0.77	intergenic
rs.3-55384577	3	55384577	A/C	0.81	intergenic
rs.4-25804581	4	25804581	A/G	0.66	intronic (*enc1*)
rs.5-10756236	5	10756236	C/T	0.81	intronic (*pigr*)
rs.5-26654404	5	26654404	G/C	0.83	intronic (*xirp1*)
rs.6-22374410	6	22374410	A/G	0.74	intergenic
rs.6-22381361	6	22381361	G/T	0.83	intergenic
rs.6-22404945	6	22404945	C/T	0.83	exonic (un)
rs.6-22406773	6	22406773	T/G	0.83	intergenic
rs.6-22478477	6	22478477	A/C	0.83	intergenic
rs.6-22500163	6	22500163	T/C	0.83	intergenic
rs.6-22503147	6	22503147	A/G	0.72	intergenic
rs.6-22503742	6	22503742	A/G	0.83	intergenic
rs.6-22504186	6	22504186	G/A	0.83	intergenic
rs.6-22507005	6	22507005	T/C	0.83	intergenic
rs.6-22546477	6	22546477	A/C	0.83	3' UTR (*hhip*)
rs.6-22551163	6	22551163	C/T	0.83	intronic (*hhip*)
rs.6-22554439	6	22554439	T/C	0.83	intronic (*hhip*)
rs.6-22556800	6	22556800	C/A	0.83	intronic (*hhip*)

SNP ID	Chr	Position	Allele	Effect	Location
rs.6-22562044	6	22562044	T/A	0.83	intronic (*hhip*)
rs.6-22564913	6	22564913	A/G	0.83	intronic (*hhip*)
rs.6-23291805	6	23291805	G/A	0.79	intergenic
rs.6-23349604	6	23349604	T/C	0.79	exonic (*klf5a*)
rs.6-23353637	6	23353637	A/T	0.79	3' UTR (*klf5a*)
rs.6-24122919	6	24122919	T/C	0.54	intergenic
rs.6-24125946	6	24125946	A/G	0.64	intergenic
rs.6-25458495	6	25458495	T/C	0.79	intronic (*mzt1*)
rs.6-26185207	6	26185207	T/C	0.79	intronic (*dis3*)
rs.6-26299737	6	26299737	A/G	0.79	intergenic
rs.6-26318387	6	26318387	G/A	0.78	intergenic
rs.6-27043452	6	27043452	A/G	0.79	intronic (*bnc2*)
rs.6-27074077	6	27074077	T/C	0.79	intronic (*bnc2*)
rs.6-27074103	6	27074103	T/A	0.79	intronic (*bnc2*)
rs.6-27098677	6	27098677	G/A	0.79	intronic (*bnc2*)
rs.6-27221880	6	27221880	A/G	0.79	intronic (*bnc2*)
rs.6-27296128	6	27296128	A/C	0.83	intronic (*bnc2*)
rs.6-27374386	6	27374386	T/C	0.79	intronic (*cntln*)
rs.6-27374447	6	27374447	T/C	0.79	intronic (*cntln*)
rs.6-27405746	6	27405746	A/T	0.79	intronic (*cntln*)
rs.6-27405993	6	27405993	A/T	0.79	intronic (*cntln*)
rs.6-27443291	6	27443291	C/T	0.77	intronic (*cntln*)
rs.6-27672625	6	27672625	A/T	0.70	intronic (*coro2aa*)
rs.6-27771987	6	27771987	G/A	0.50	intronic (*arhgef8*)
rs.6-27782483	6	27782483	A/G	0.58	intergenic
rs.6-44745870	6	44745870	T/A	0.78	intronic (*pard3ba*)
rs.6-44788061	6	44788061	G/C	0.76	intronic (*pard3ba*)
rs.6-44788142	6	44788142	T/G	0.76	intronic (*pard3ba*)
rs.6-44788251	6	44788251	T/C	0.76	intronic (*pard3ba*)
rs.7-23831209	7	23831209	C/G	0.81	exonic (un)
rs.7-23831219	7	23831219	C/A	0.81	exonic (un)
rs.7-23831258	7	23831258	A/G	0.79	exonic (un)
rs.9-27437449	9	27437449	A/T	0.77	intronic (un)

SNP ID	Chr	Position	Allele	Effect	Location
rs.12-9314173	12	9314173	G/A	0.51	exonic (*znf271*)
rs.12-27641629	12	27641629	G/A	0.80	intronic (*kcnh8*)
rs.14-29442705	14	29442705	T/A	-0.81	intergenic
rs.15-36268252	15	36268252	G/T	0.76	intronic (*grid1a*)
rs.16-13240372	16	13240372	G/A	0.71	exonic (un)
rs.16-16389188	16	16389188	G/C	0.74	intronic (*mbtps1*)
rs.17-27400821	17	27400821	T/C	0.58	intronic (*rbfox3a*)
rs.17-36307674	17	36307674	T/C	0.78	intronic (un)
rs.18-12110368	18	12110368	T/G	0.73	intronic (*lingo2*)
rs.19-110807	19	110807	G/T	0.71	intronic (un)
rs.20-1485091	20	1485091	T/C	0.71	intergenic
rs.20-31965512	20	31965512	C/A	-0.42	intronic (*esco1*)
rs.23-28457737	23	28457737	T/A	-0.64	exonic (*dnajc16*)
rs.23-28458362	23	28458362	T/C	-0.60	exonic (*dnajc16*)
rs.25-27346992	25	27346992	A/G	0.80	intronic (*wwox*)
rs.25-27347225	25	27347225	A/G	0.80	intronic (*wwox*)
rs.25-27349254	25	27349254	G/A	0.80	intronic (*wwox*)
rs.25-27349773	25	27349773	G/A	0.80	intronic (*wwox*)

SNP ID, SNPs are denoted by chromosome and physical position; Chr, chromosome; Position, physical position; Allele, alternative/reference; Effect, effect size of each SNP; Location, the location on the gene.

Supplementary Table S3. Candidate genes associated with sex in *Misgurnus anguillicaudatus*.

Candidate gene	SNP ID	Chr	Start	End	Description
cnpy4	rs.1-20849158	1	20798754	20802374	protein canopy 4
capn5	rs.1-20849158	1	20803048	20854633	calpain-5
LOC129439018	rs.1-20849158	1	20862071	20864985	transmembrane protein 272
zgc_103586	rs.1-20849158	1	20871770	20874753	zgc:103586
slc3a2	rs.1-20849158	1	20890057	20900040	4F2 cell-surface antigen heavy chain
LOC129448867	rs.1-48520965	1	48532006	48814681	pyruvate carboxylase, mitochondrial-like
znf845	rs.1-56459438	2	56393815	56616031	zinc finger protein 845
LOC129422495	rs.1-56459438	2	56405247	56411954	transposable element
zbed1	rs.1-56459438	2	56420764	56424160	E3 SUMO-protein ligase ZBED1
LOC129422842	rs.1-56459438	2	56438953	56450487	uncharacterized
LOC129422133	rs.1-56459438	2	56445429	56449160	uncharacterized
mef2d	rs.1-56459438	2	56478730	56482577	myocyte-specific enhancer factor 2D
g2e3	rs.1-56459438	2	56482783	56483877	G2/M phase-specific E3 ubiquitin-protein ligase
LOC129423108	rs.1-56459438	2	56488338	56489704	uncharacterized
golga4	rs.1-56459438	2	56494146	56497573	golgin subfamily A member 4
LOC129422091	rs.1-56459438	2	56503843	56508348	uncharacterized

Candidate gene	SNP ID	Chr	Start	End	Description
pik3cb	rs.3-495588, rs.3-495952, rs.3-502503, rs.3-503456, rs.3-503515, rs.3-504516, rs.3-505043, rs.3-506044	3	453490	511836	phosphatidylinositol 4,5-bisphosphate 3-kinase catalytic subunit beta
foxl2a	rs.3-495588, rs.3-495952, rs.3-502503, rs.3-503456, rs.3-503515, rs.3-504516, rs.3-505043, rs.3-506044	3	524897	527577	forkhead box protein L2a
atm	rs.3-1161899, rs.3-1162173	3	1126391	1186627	serine-protein kinase ATM
nop53	rs.3-1161899, rs.3-1162173	3	1186999	1191876	ribosome biogenesis protein NOP53
gjd2	rs.3-1161899, rs.3-1162173	3	1201235	1225227	gap junction delta-2 protein
LOC129438392	rs.3-33338471, rs.3-33338533	3	33384503	33864682	uncharacterized
c3ar1	rs.3-33338471, rs.3-33338533	3	33284585	33288817	c3a anaphylatoxin chemotactic receptor
LOC129439025	rs.3-55384577	3	55404602	55418427	protein NLRC3-like
aif1l	rs.4-25804581	4	25740529	25766744	allograft inflammatory factor 1
gfm2	rs.4-25804581	4	25770809	25786534	ribosome-releasing factor 2, mitochondrial
hexb	rs.4-25804581	4	25786502	25797154	beta-hexosaminidase subunit beta
enc1	rs.4-25804581	4	25799298	25811806	ectoderm-neural cortex protein 1
pigr	rs.5-10756236	5	10732499	10772627	polymeric immunoglobulin receptor

Candidate gene	SNP ID	Chr	Start	End	Description
tgfbr1a	rs.5-26654404	5	26581010	26618019	TGF-beta receptor type-1a
vopp1	rs.5-26654404	5	26627139	26631201	vesicular, overexpressed in cancer, prosurvival protein 1
gorasp1a	rs.5-26654404	5	26631340	26638330	Golgi reassembly-stacking protein 1a
csrnp1a	rs.5-26654404	5	26638597	26645175	cysteine/serine-rich nuclear protein 1
xirp1	rs.5-26654404	5	26650857	26671263	xin actin-binding repeat-containing protein 1
slc22a13a	rs.5-26654404	5	26677641	26684043	solute carrier family 22 member 13
oxsr1a	rs.5-26654404	5	26686657	26703504	serine/threonine-protein kinase OSR1
smad1	rs.6-22374410	6	22299303	22329377	mothers against decapentaplegic homolog 1
LOC129444446	rs.6-22381361, rs.6-22404945 rs.6-22406773	6	22392707	22397888	uncharacterized
LOC129445423	rs.6-22404945, rs.6-22406773	6	22403737	22406114	uncharacterized
hhip	rs.6-22500163, rs.6-22503147 rs.6-22503742, rs.6-22504186 rs.6-22507005, rs.6-22546477 rs.6-22551163, rs.6-22554439 rs.6-22556800, rs.6-22562044 rs.6-22564913	6	22545621	22583959	hedgehog-interacting protein
tctp	rs.6-23291805	6	23236026	23257691	translationally-controlled tumor protein homolog
gtf2f2	rs.6-23291805	6	23273713	23283989	general transcription factor IIF subunit 2

Candidate gene	SNP ID	Chr	Start	End	Description
gpalpp1	rs.6-23291805	6	23295585	23295585	GPALPP motifs-containing protein 1
pibf1	rs.6-23291805, rs.6-23349604	6	23299068	23335172	progesterone-induced-blocking factor 1
klf5a	rs.6-23349604, rs.6-23353637	6	23347355	23354604	krueppel-like factor 5
klf12a	rs.6-23349604, rs.6-23353637	6	23386563	23599397	krueppel-like factor 12a
kctd12.2	rs.6-24122919, rs.6-24125946	6	24074574	24076344	BTB/POZ domain-containing protein KCTD12.2
slc5a7a	rs.6-24122919, rs.6-24125946	6	24171538	24180878	high affinity choline transporter 1
mzt1	rs.6-25458495	6	25450631	25459790	mitotic-spindle organizing protein 1
bora	rs.6-25458495	6	25460673	25478857	protein aurora borealis
dis3	rs.6-26185207	6	25966189	26206787	exosome complex exonuclease RRP44
pspc1	rs.6-26185207	6	26207598	26244913	Paraspeckle component 1
LOC129444536	rs.6-26299737, rs.6-26318387	6	26271130	26278982	uncharacterized
LOC129438891	rs.6-26299737, rs.6-26318387	6	26282800	26284840	uncharacterized protein K02A2.6
chst10	rs.6-26318387	6	26335789	26386691	carbohydrate sulfotransferase 10
bnc2	rs.6-27043452, rs.6-27074077<br>rs.6-27074103, rs.6-27098677<br>rs.6-27221880, rs.6-27296128<br>rs.6-27374386, rs.6-27374447	6	26940701	27335090	zinc finger protein basonuclin-2
LOC129445444	rs.6-27296128	6	27275628	27278759	uncharacterized

Candidate gene	SNP ID	Chr	Start	End	Description
cntln	rs.6-27374386, rs.6-27374447, rs.6-27405746, rs.6-27405993, rs.6-27443291	6	27358121	27503698	centlein
spag5	rs.6-27672625	6	27617716	27634321	sperm-associated antigen 5
trmo	rs.6-27672625	6	27634630	27643520	tRNA (adenine(37)-N6)-methyltransferase
hemgn	rs.6-27672625	6	27643640	27645974	hemogen
anp32b	rs.6-27672625	6	27649208	27657785	acidic leucine-rich nuclear phosphoprotein 32 family member B
coro2aa	rs.6-27672625	6	27660883	27686392	coronin-2A
uso1	rs.6-27672625, rs.6-27771987	6	27694353	27734535	general vesicular transport factor p115
g3bp2	rs.6-27771987, rs.6-27782483	6	27744863	27757942	ras GTPase-activating protein-binding protein 2
ints12	rs.6-27771987, rs.6-27782483	6	27758012	27763750	integrator complex subunit 12
arhgef38	rs.6-27771987, rs.6-27782483	6	27764062	27780305	rho guanine nucleotide exchange factor 38
ppa2	rs.6-27771987, rs.6-27782483	6	27788218	27793028	inorganic pyrophosphatase 2, mitochondrial
tet2	rs.6-27771987, rs.6-27782483	6	27798184	27825560	methylcytosine dioxygenase TET2
pard3ba	rs.6-44745870, rs.6-44788061, rs.6-44788142, rs.6-44788251	6	44696391	44903211	par-3 family cell polarity regulator beta a
LOC129447285	rs.7-23831209, rs.7-23831219, rs.7-23831258	7	23791936	23802275	uncharacterized

Candidate gene	SNP ID	Chr	Start	End	Description
LOC129447292	rs.7-23831209, rs.7-23831219, rs.7-23831258	7	23804867	23814465	gastrula zinc finger protein XlCGF8.2DB-like
hmgxb3	rs.7-23831209, rs.7-23831219, rs.7-23831258	7	23824764	23831713	HMG domain-containing protein 3
LOC129447289	rs.7-23831209, rs.7-23831219, rs.7-23831258	7	23867511	23871344	uncharacterized
tc1a	rs.7-23831209, rs.7-23831219, rs.7-23831258	7	23873301	23884738	transposable element Tc1 transposase
LOC129450968	rs.9-27437449	9	27396234	27398127	uncharacterized
pgbd3	rs.9-27437449	9	27419485	27422742	piggyBac transposable element-derived protein 3
znf883	rs.12-9314173	12	9266196	9270078	zinc finger protein 883
znf569	rs.12-9314173	12	9274721	9281407	zinc finger protein 569
gin1	rs.12-9314173	12	9284212	9291816	Gypsy retrotransposon integrase-like protein 1
znf271	rs.12-9314173	12	9295698	9314343	zinc finger protein 271
znf426	rs.12-9314173	12	9358283	9359864	zinc finger protein 426
kcnh8	rs.12-27641629	12	27586207	27665837	potassium voltage-gated channel subfamily H member 8
satb1	rs.12-27641629	12	27589075	27598549	DNA-binding protein SATB1
rab5a	rs.12-27641629	12	27691085	27696320	ras-related protein Rab-5A
nlrc3	rs.14-29442705	14	29401012	29408819	NLR family CARD domain-containing protein 3

Candidate gene	SNP ID	Chr	Start	End	Description
nlrp3	rs.14-29442705	14	29412849	29441078	NACHT, LRR and PYD domains-containing protein 3-like
ubr2	rs.14-29442705	14	29444602	29480607	E3 ubiquitin-protein ligase UBR2
trpv6	rs.14-29442705	14	29481263	29496118	transient receptor potential cation channel subfamily V member 6
grid1a	rs.15-36268252	15	35811191	36292394	glutamate receptor ionotropic, delta-1
wapl	rs.15-36268252	15	36295216	36323270	wings apart-like protein homolog
eif4g2	rs.16-13240372	16	13191752	13207530	Eukaryotic translation initiation factor 4 gamma 2
myh9	rs.16-13240372	16	13209739	13215907	myosin-9
lyve1	rs.16-13240372	16	13217945	13222483	lymphatic vessel endothelial hyaluronic acid receptor 1
rnf141	rs.16-13240372	16	13222541	13233016	RING finger protein 141
LOC129419814	rs.16-13240372	16	13238316	13254881	uncharacterized
necab2	rs.16-16389188	16	16279325	16370928	N-terminal EF-hand calcium-binding protein 2
slc38a8	rs.16-16389188	16	16373673	16378137	putative sodium-coupled neutral amino acid transporter 8a
mbtps1	rs.16-16389188	16	16383038	16406320	membrane-bound transcription factor site-1 protease
fan1	rs.16-16389188	16	16406939	16417846	Fanconi-associated nuclease 1
pskh1	rs.16-16389188	16	16418239	16431525	serine/threonine-protein kinase H1 homolog
rbfox3a	rs.17-27400821	17	27112921	27421618	RNA binding protein fox-1 homolog 3

Candidate gene	SNP ID	Chr	Start	End	Description
LOC129421345	rs.17-36307674	17	36190890	36339978	uncharacterized
LOC129421344	rs.17-36307674	17	36258095	36368836	uncharacterized
LOC129421346	rs.17-36307674	17	36311184	36312998	uncharacterized
LOC129421347	rs.17-36307674	17	36333089	36338590	uncharacterized
lingo2	rs.18-12110368	18	11938733	12294412	leucine-rich repeat and immunoglobulin domain-containing nogo receptor-interacting protein 2
LOC129423929	rs.19-110807	19	58302	61336	uncharacterized
LOC129424252	rs.19-110807	19	80649	81956	uncharacterized
LOC129423042	rs.19-110807	19	104060	111739	uncharacterized
LOC129424253	rs.19-110807	19	123522	144871	uncharacterized
muc2	rs.19-110807	19	151671	186326	mucin-2-like
srrm5	rs.19-110807	19	155802	177116	serine/arginine repetitive matrix protein 5
LOC129425947	rs.20-1485091	20	1476551	1479212	uncharacterized
obscnb	rs.20-31965512	20	31851696	31917711	obscurin
gnal2	rs.20-31965512	20	31918248	31952178	guanine nucleotide binding protein (G protein), alpha activating activity polypeptide, olfactory type 2
tmem14cb	rs.20-31965512	20	31953335	31955432	transmembrane protein 14Cb
pak1ip1	rs.20-31965512	20	31955746	31960210	p21-activated protein kinase-interacting protein 1

Candidate gene	SNP ID	Chr	Start	End	Description
esco1	rs.20-31965512	20	31961268	31970146	N-acetyltransferase ESCO1
rbbp8	rs.20-31965512	20	31970443	31980232	DNA endonuclease RBBP8
tmem241	rs.20-31965512	20	31980858	31989844	transmembrane protein 241
riok3	rs.20-31965512	20	31991291	32002119	serine/threonine-protein kinase RIO3
mylk	rs.20-31965512	20	32008671	32021875	myosin light chain kinase, smooth muscle
zmp_0000001088	rs.23-28457737, rs.23-28458362	23	28397126	28417744	trypsin
LOC129431529	rs.23-28457737, rs.23-28458362	23	28426809	28432316	trypsin I-P1-like
cdaa	rs.23-28457737, rs.23-28458362	23	28432892	28434465	cytidine deaminase a
pink1	rs.23-28457737, rs.23-28458362	23	28436852	28444250	serine/threonine-protein kinase PINK1, mitochondrial
agmat	rs.23-28457737, rs.23-28458362	23	28444439	28449634	agmatinase, mitochondrial
dnajc16	rs.23-28457737, rs.23-28458362	23	28452763	28464111	dnaJ homolog subfamily C member 16
tor1a	rs.23-28457737, rs.23-28458362	23	28476831	28494146	torsin-1A-like
LOC129422150	rs.23-28457737, rs.23-28458362	23	28483094	28485710	uncharacterized
wwox	rs.25-27346992, rs.25-27347225, rs.25-27349254, rs.25-27349773	25	27259531	27351062	WW domain-containing oxidoreductase
mafb	rs.25-27346992, rs.25-27347225, rs.25-27349254, rs.25-27349773	25	27388241	27454151	transcription factor MafB

SNP ID, SNPs are denoted by chromosome and physical position; Chr, chromosome; Start and End, physical location of the start and end of the gene in the reference genome.

Supplementary Table S4. Gene sequence information used for qRT-PCR analysis of *Misgurnus anguillicaudatus*.

Gene	GenBank accession No.
cnpy4	XM_055197891.1
foxl2a	XM_055195582.1
bnc2	XM_055205283.1
hemgn	XM_055205301.1
pik3cb	XM_055195577.1
tgfbr1a	XM_055202256.1
smad1	XM_055205126.1
hhip	XM_055205129.1
chst10	XM_055205277.1
znf271	XM_055219011.1
capn5	XM_055197849.1
bora	XM_055205236.1
cntln	XM_055205290.1
spag5	XM_055205265.1
pard3ba	XM_055206148.1
ubr2	XM_055170867.1
esco1	XM_055182832.1
atm	XM_055195612.1
enc1	XM_055199623.1
klf5a	XM_055205170.2
dis3	XM_055205264.1
pspc1	XM_055205294.1
dnajc16	XM_055188510.1
wwox	XM_055191886.1

Supplementary Table S5. Primers used for mRNA expression analysis of candidate genes and amplification of sex-specific SNP marker.

Reactions	Primers	Description	Sequence (5' to 3')
qRT-PCR	*cnpy4*-F	Forward primer for *cnpy4* qRT-PCR	TTGGACACTGGCAAGCGAAG
	cnpy4-R	Reverse primer for *cnpy4* qRT-PCR	ATGGCTCATCCCACAGTTCA
	foxl2a-F	Forward primer for *foxl2a* qRT-PCR	CTCACGCTGTCCGGTATCTA
	foxl2a-R	Reverse primer for *foxl2a* qRT-PCR	GTCTCTTCATGCGCCGTCTC
	bnc2-F	Forward primer for *bnc2* qRT-PCR	CAGCAAGGGGCAGAAGATGA
	bnc2-R	Reverse primer for *bnc2* qRT-PCR	ACATTGGTGGGCTTGTTCCA
	hemgn-F	Forward primer for *hemgn* qRT-PCR	CCACGGAAGTCGAGACAACA
	hemgn-R	Reverse primer for *hemgn* qRT-PCR	TGCTGACACTGAGGCTTGAC
	pik3cb-F	Forward primer for *pik3cb* qRT-PCR	GGAGAGCACCATCCAACACA
	pik3cb-R	Reverse primer for *pik3cb* qRT-PCR	GCCTGCTTTCATCCTCCAGT
	tgfbr1a-F	Forward primer for *tgfbr1a* qRT-PCR	GTGCCCATCTGTGTGCTGAT
	tgfbr1a-R	Reverse primer for *tgfbr1a* qRT-PCR	CCCTTTCCTATGCTCTCCTGC
	smad1-F	Forward primer for *smad1* qRT-PCR	GCAAATGCCCCTCCCTTTAC
	smad1-R	Reverse primer for *smad1* qRT-PCR	CATATAAGCAGGTGGGGGCG
	hhip-F	Forward primer for *hhip* qRT-PCR	AAAGGGCGGAGATGAAAGGG
	hhip-R	Reverse primer for *hhip* qRT-PCR	GGAGAATGTGGTCGTGTGGT
	chst10-F	Forward primer for *chst10* qRT-PCR	CCCCAGCCATCATCCGAAAA
	chst10-R	Reverse primer for *chst10* qRT-PCR	ACAAGGAGCACACAGGTCAG
	znf271-F	Forward primer for *znf271* qRT-PCR	AGAAAGAGGTGGCGTTACTGG
	znf271-R	Reverse primer for *znf271* qRT-PCR	AGTTTAGAGTCCAGCAGTGGC

Reactions	Primers	Description	Sequence (5' to 3')
qRT-PCR	*capn5*-F	Forward primer for *capn5* qRT-PCR	GGATGGAACGCTGCTCTTCT
	capn5-R	Reverse primer for *capn5* qRT-PCR	GGGTTCAGAGACACCACCAG
	bora-F	Forward primer for *bora* qRT-PCR	ATGAAGCTCTTGTGCCCAGC
	bora-R	Reverse primer for *bora* qRT-PCR	TGGCGGTGAATATCCTCTGG
	cntln-F	Forward primer for *cntln* qRT-PCR	GACTCGCTTCTCCAGCAACT
	cntln-R	Reverse primer for *cntln* qRT-PCR	AAGCTGTTTGACTTGGGCCT
	spag5-F	Forward primer for *spag5* qRT-PCR	CGAGAGGTTGCTGATTTGCG
	spag5-R	Reverse primer for *spag5* qRT-PCR	TGGCACGATTTTCCTCGGTT
	pard3ba-F	Forward primer for *pard3ba* qRT-PCR	TTGGAGGTGCTTCCTGTTGC
	pard3ba-R	Reverse primer for *pard3ba* qRT-PCR	TGTCCAGGTTGACTGGCTTG
	ubr2-F	Forward primer for *ubr2* qRT-PCR	GGTTGTGGGTGAGCGATACA
	ubr2-R	Reverse primer for *ubr2* qRT-PCR	TCTCCATGCCGGTCTCCTTA
	esco1-F	Forward primer for *esco1* qRT-PCR	GAGGATGGAGGAAAGCCCTG
	esco1-R	Reverse primer for *esco1* qRT-PCR	CCTCGACTGTGGCAGATGTT
	atm-F	Forward primer for *atm* qRT-PCR	ACTCCTCATCTCACTCCGCT
	atm-R	Reverse primer for *atm* qRT-PCR	GACCAGGGATTCTTGCACCA
	enc1-F	Forward primer for *enc1* qRT-PCR	CCAGTCATGCGCTTTTCCTG
	enc1-R	Reverse primer for *enc1* qRT-PCR	CCTTGGACACACCGTTCTCA
	klf5a-F	Forward primer for *klf5a* qRT-PCR	CAGCCTCACTCGCATCTCAA
	klf5a-R	Reverse primer for *klf5a* qRT-PCR	CAGGTCTGGGTTCGTCCTTC
	dis3-F	Forward primer for *dis3* qRT-PCR	ACCAATGAGCATCACAGGGG

Reactions	Primers	Description	Sequence (5'to 3')
qRT-PCR	*dis3*-R	Reverse primer for *dis3* qRT-PCR	CGATCATGGGTTGCTTTGGC
	pspc1-F	Forward primer for *pspc1* qRT-PCR	AGGCTGAAATGGAGGCTGTC
	pspc1-R	Reverse primer for *pspc1* qRT-PCR	CGTCTTTCTTCCTCGTGCCT
	dnajc16-F	Forward primer for *dnajc16* qRT-PCR	GCATGAGCTGAACAAGCCAC
	dnajc16-R	Reverse primer for *dnajc16* qRT-PCR	ATAGTCGGCGCATACGTGTT
	wwox-F	Forward primer for *wwox* qRT-PCR	ACATATTTTGACCCCCGGCA
	wwox-R	Reverse primer for *wwox* qRT-PCR	GCACGACTTTTGTTTCGGCA
	ef1a-F	Forward primer for *ef1a* qRT-PCR	CTGGAGACAGCAAGAACGACCCAC C
	ef1a-R	Reverse primer for *ef1a* qRT-PCR	GGCAATCCAGCACTGGAGCATAGCC
Sex marker	sex-F	Forward primer for sex-specific SNPs	TTTCAAAATGAGCATCTGT
	sex-R	Reverse primer for sex-specific SNPs	GAGGTTATTTTAGTTTTGGAT

Supplementary Table S6. Haplotype analysis of SNPs in *pik3cb*, *hhip*, and *cntln*.

Gene	Hap ID	Genotype	Male (%)	Female (%)	*P*-value	OR (95% CI)
pik3cb	Hap1	CT	70	95	0.0005	8.143 (2.312 to 27.09)
		TA	28.33	0	<0.0001	0.000 (0.000 to 0.1681)
		CA	1.67	5	0.6186	3.105 (0.4482 to 40.93)
	Hap2	GC	81.67	80	>0.9999	1.114 (0.4621 to 2.758)
		AT	15	16.67	>0.9999	1.133 (0.4428 to 2.876)
hhip	Hap1	AT	75	100	<0.0001	Infinity (4.904 to Infinity)
		GC	23.33	0	<0.0001	0.000 (0.000 to 0.2266)
		AC	1.67	0	>0.9999	0.000 (0.000 to 9.000)
	Hap2	AG	53.33	81.67	0.0016	3.898 (1.705 to 8.559)
		CT	46.67	15	0.0003	0.2017 (0.08817 to 0.4908)
		CG	0	3.33	0.4958	Infinity (0.4650 to Infinity)
cntln	Hap1	GC	76.67	100	<0.0001	Infinity (4.413 to Infinity)
		CT	21.67	0	0.0001	0.000 (0.000 to 0.2538)
		GT	1.67	0	>0.9999	0.000 (0.000 to 9.000)
	Hap2	CT	36.67	53.33	0.0982	1.974 (0.9352 to 4.031)
		GC	60	41.67	0.0674	0.4762 (0.2340 to 1.000)
		CC	3.33	5.00	>0.9999	1.526 (0.3015 to 8.820)
	Hap3	TA	83.33	70.00	0.1300	0.4667 (0.1910 to 1.097)
		GG	16.67	20.00	0.8140	1.250 (0.5057 to 3.305)
		TG	0	10.00	0.0274	Infinity (1.456 to Infinity)

Gene	Hap ID	Genotype	Male (%)	Female (%)	*P*-value	OR (95% CI)
cntln	Hap4	TGC	76.67	100	<0.0001	Infinity (4.413 to Infinity)
		AAG	21.67	0	0.0001	0.000 (0.000 to 0.2538)
		AGC	1.67	0	>0.9999	0.000 (0.000 to 9.000)

Male (%), genotype proportion in males; Female (%), genotype proportion in females; OR, odds ratio; CI, confidence interval.

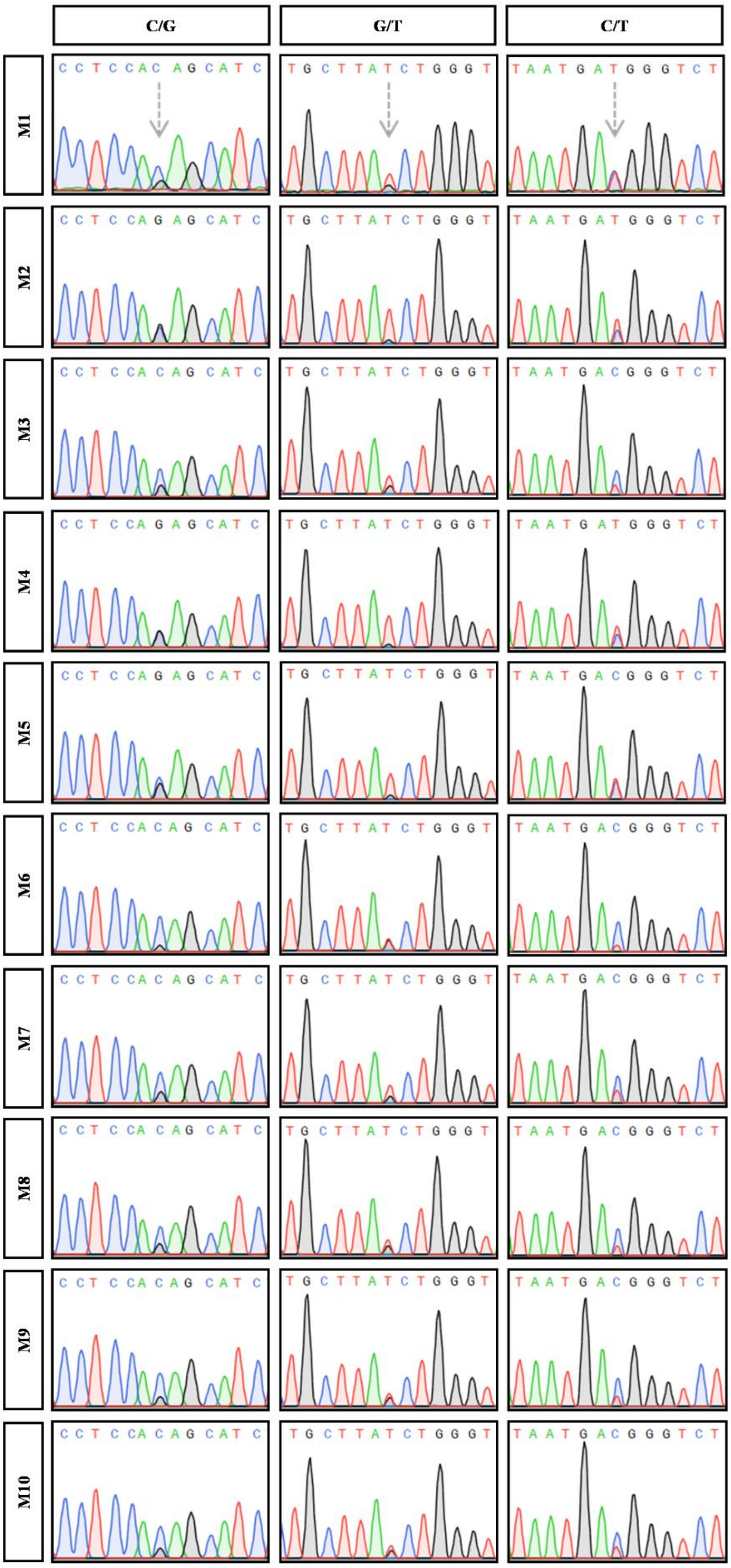

Supplementary Figure S1. Sanger sequencing map of PCR-amplified products at the sex-specific SNP loci for ten physiological males in population 1. M1-M10 represent physiological male individuals. The arrow points to the peak map corresponding to the SNP loci.

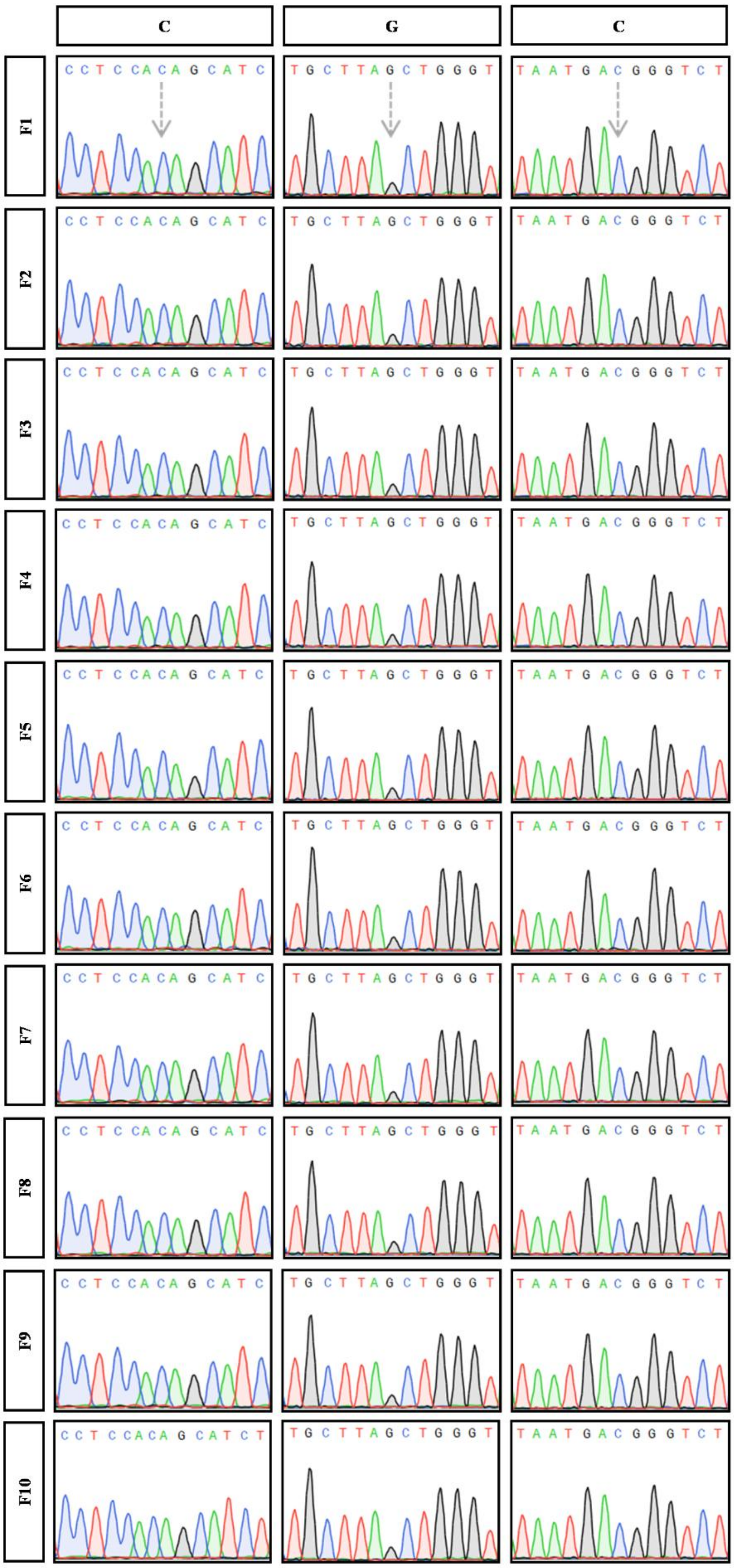

Supplementary Figure S2. Sanger sequencing map of PCR-amplified products at the sex-specific SNP loci for ten physiological females in population 1. F1-F10 represent physiological female individuals. The arrow points to the peak map corresponding to the SNP loci.

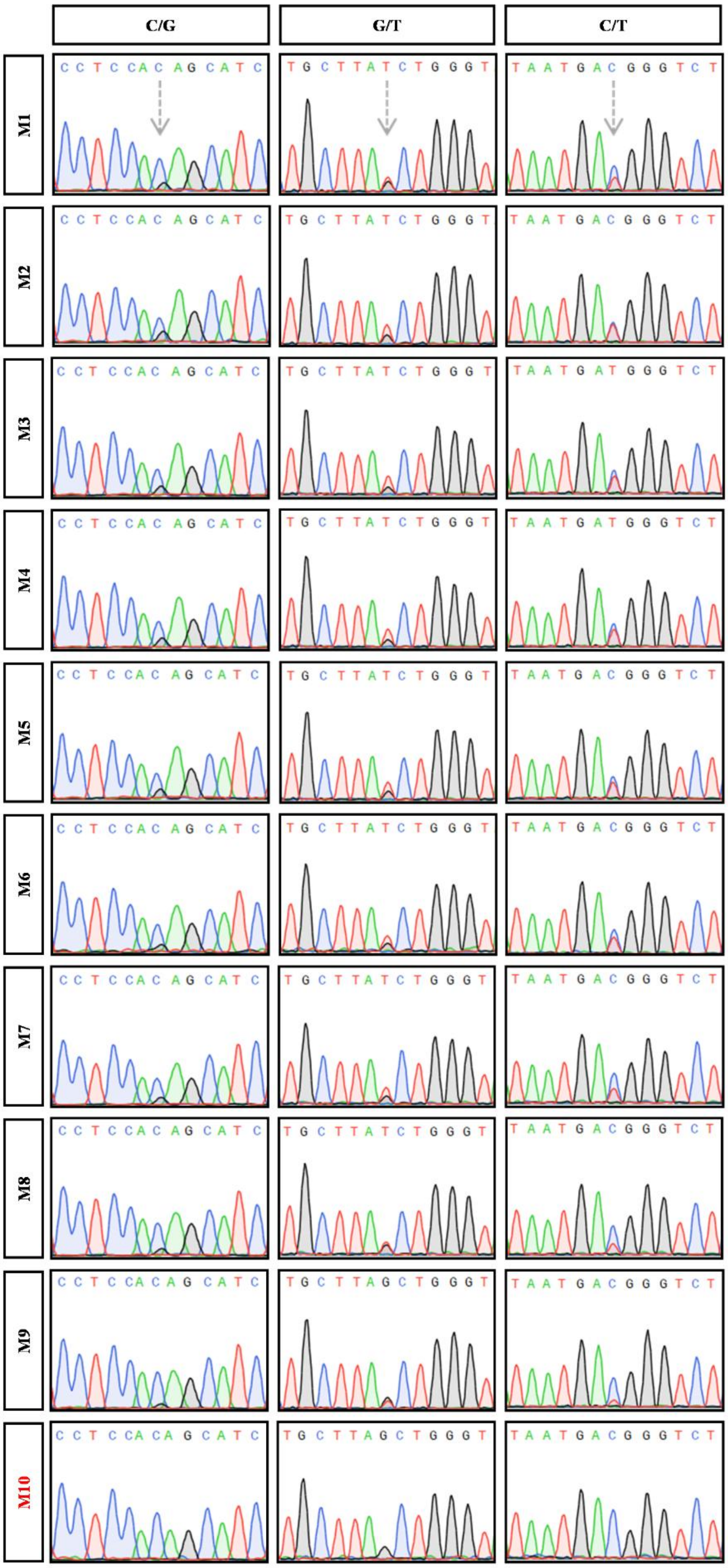

Supplementary Figure S3. Sanger sequencing map of PCR-amplified products at the sex-specific SNP loci for ten physiological males in population 2. M1-M10 represent physiological male individuals, of which the red font indicates that the genetic sex identified by the sex-specific marker does not match the physiological sex.

The arrow points to the peak map corresponding to the SNP loci.

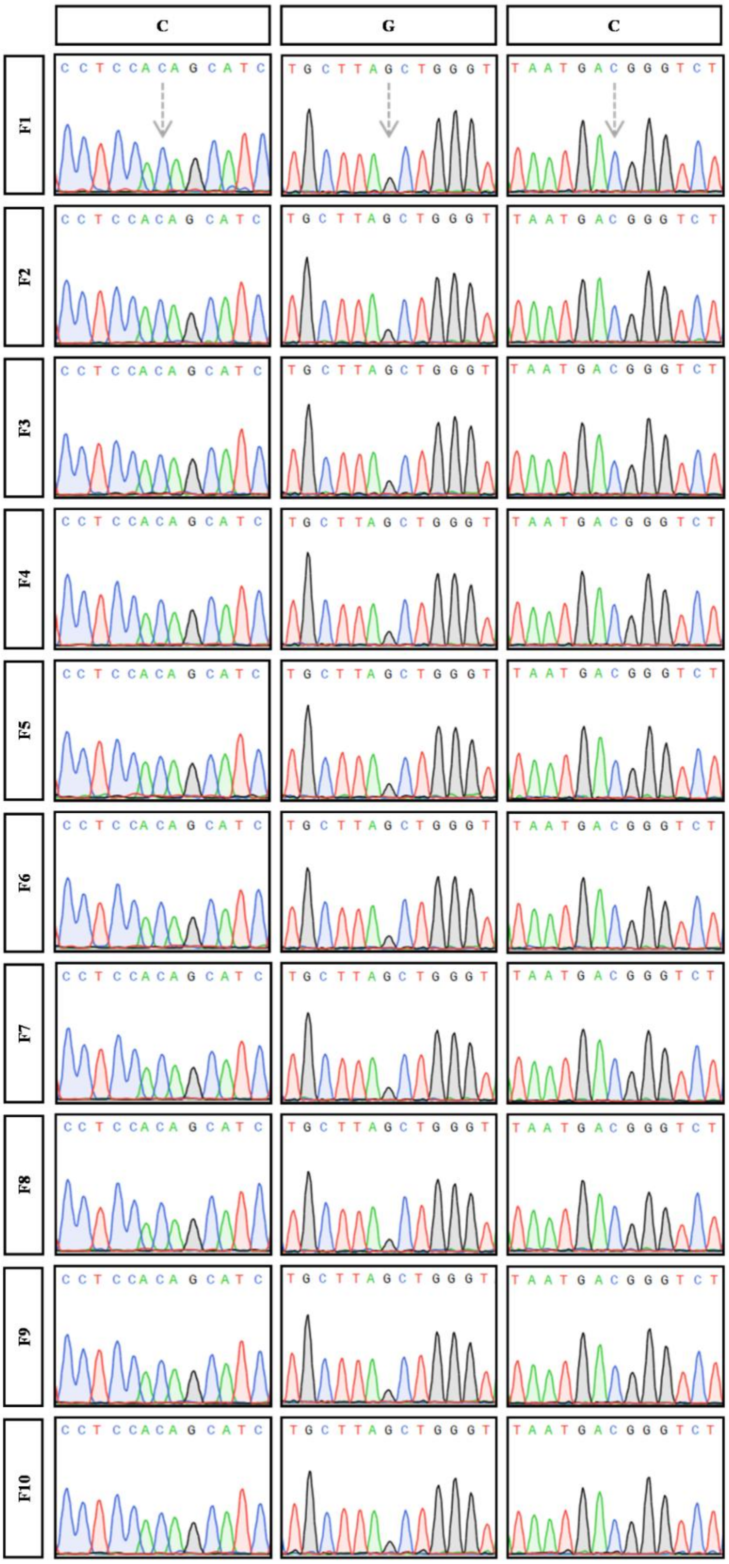

Supplementary Figure S4. Sanger sequencing map of PCR-amplified products at the sex-specific SNP loci for ten physiological females in population 2. F1-F10 represent physiological female individuals. The arrow points to the peak map corresponding to the SNP loci.

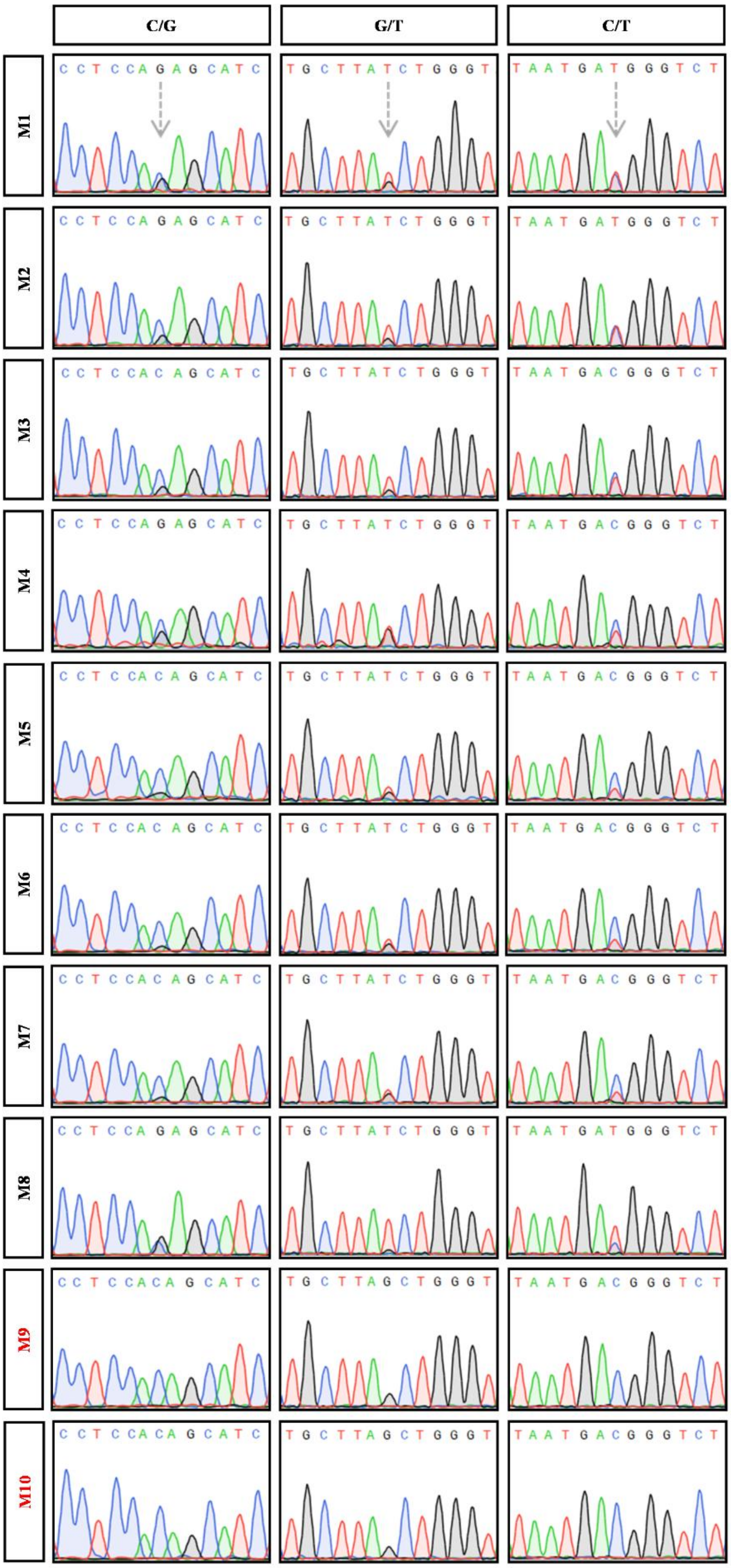

Supplementary Figure S5. Sanger sequencing map of PCR-amplified products at the sex-specific SNP loci for ten physiological males in population 3. M1-M10 represent physiological male individuals, of which the red fonts indicate that the genetic sex identified by the sex-specific marker does not match the physiological sex. The arrow points to the peak map corresponding to the SNP loci.

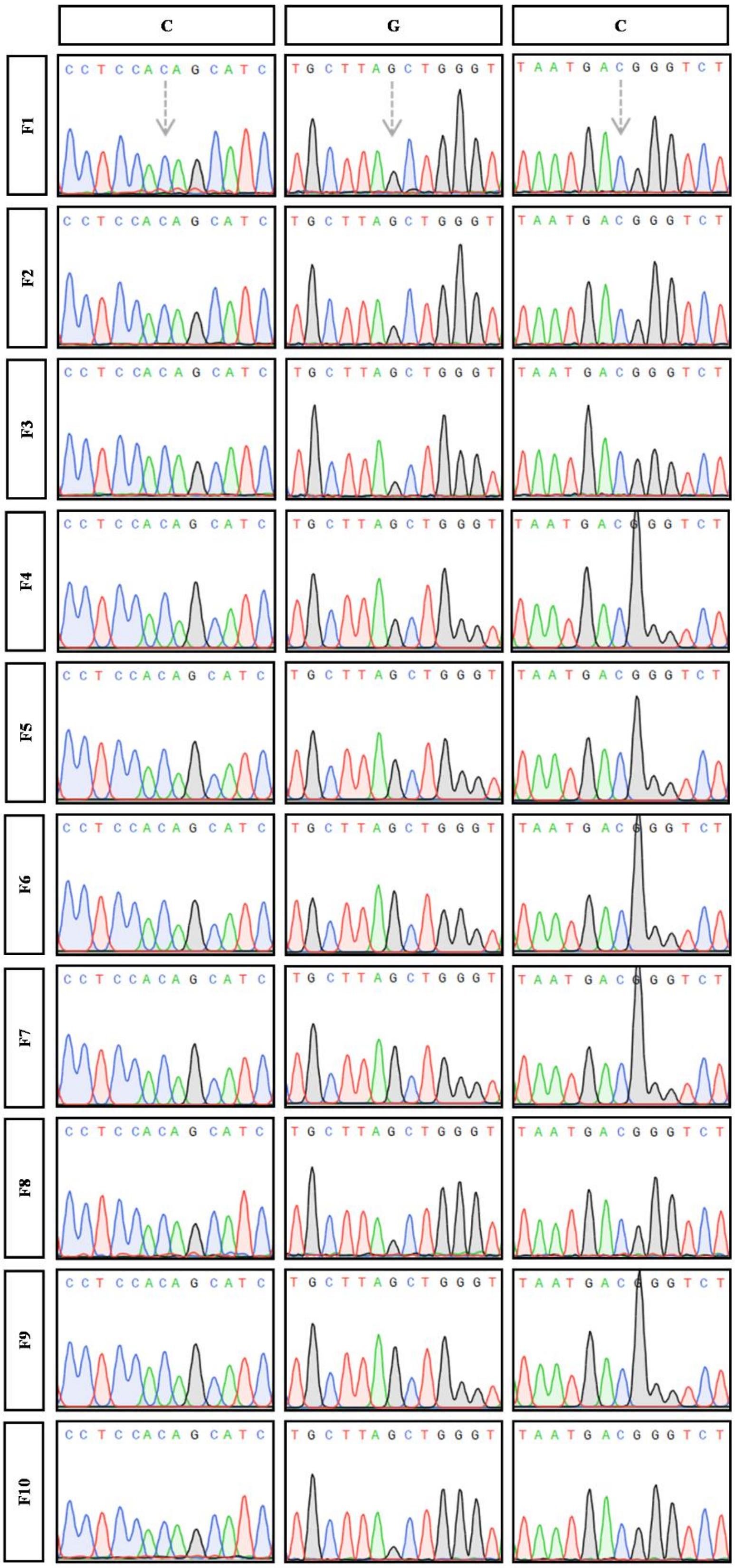

Supplementary Figure S6. Sanger sequencing map of PCR-amplified products at the sex-specific SNP loci for ten physiological females in population 3. F1-F10 represent physiological female individuals. The arrow points to the peak map corresponding to the SNP loci.
